# DNA 5-hydroxymethylcytosine in pediatric central nervous system tumors may impact tumor classification and is a positive prognostic marker

**DOI:** 10.1186/s13148-021-01156-9

**Published:** 2021-09-19

**Authors:** Nasim Azizgolshani, Curtis L. Petersen, Youdinghuan Chen, Joshua J. Levy, Lucas A. Salas, Laurent Perreard, Lananh N. Nguyen, Brock C. Christensen

**Affiliations:** 1 Department of Molecular and Systems Biology, Geisel School of Medicine at Dartmouth, Lebanon, NH 03756 USA; 2 The Dartmouth Institute for Health Policy and Clinical Practice, Dartmouth College, Lebanon, NH 03756 USA; 3 Department of Epidemiology, Geisel School of Medicine at Dartmouth, Lebanon, NH 03756 USA; 4 Department of Pathology, Dartmouth-Hitchcock Medical Center, Lebanon, NH 03756 USA; 5 Department of Laboratory Medicine and Pathobiology, University of Toronto, Toronto, ON Canada

**Keywords:** Epigenetics, 5-Hydroxymethylcytosine, Pediatric CNS/brain tumors, Cancer, Methylation

## Abstract

**Background:**

Nucleotide-specific 5-hydroxymethylcytosine (5hmC) remains understudied in pediatric central nervous system (CNS) tumors. 5hmC is abundant in the brain, and alterations to 5hmC in adult CNS tumors have been reported. However, traditional approaches to measure DNA methylation do not distinguish between 5-methylcytosine (5mC) and its oxidized counterpart 5hmC, including those used to build CNS tumor DNA methylation classification systems. We measured 5hmC and 5mC epigenome-wide at nucleotide resolution in glioma, ependymoma, and embryonal tumors from children, as well as control pediatric brain tissues using tandem bisulfite and oxidative bisulfite treatments followed by hybridization to the Illumina Methylation EPIC Array that interrogates over 860,000 CpG loci.

**Results:**

Linear mixed effects models adjusted for age and sex tested the CpG-specific differences in 5hmC between tumor and non-tumor samples, as well as between tumor subtypes. Results from model-based clustering of tumors was used to test the relation of cluster membership with patient survival through multivariable Cox proportional hazards regression. We also assessed the robustness of multiple epigenetic CNS tumor classification methods to 5mC-specific data in both pediatric and adult CNS tumors. Compared to non-tumor samples, tumors were hypohydroxymethylated across the epigenome and tumor 5hmC localized to regulatory elements crucial to cell identity, including transcription factor binding sites and super-enhancers. Differentially hydroxymethylated loci among tumor subtypes tended to be hypermethylated and disproportionally found in CTCF binding sites and genes related to posttranscriptional RNA regulation, such as *DICER1*. Model-based clustering results indicated that patients with low 5hmC patterns have poorer overall survival and increased risk of recurrence. Our results suggest 5mC-specific data from OxBS-treated samples impacts methylation-based tumor classification systems giving new opportunities for further refinement of classifiers for both pediatric and adult tumors.

**Conclusions:**

We identified that 5hmC localizes to super-enhancers, and genes commonly implicated in pediatric CNS tumors were differentially hypohydroxymethylated. We demonstrated that distinguishing methylation and hydroxymethylation is critical in identifying tumor-related epigenetic changes. These results have implications for patient prognostication, considerations of epigenetic therapy in CNS tumors, and for emerging molecular neuropathology classification approaches.

**Supplementary Information:**

The online version contains supplementary material available at 10.1186/s13148-021-01156-9.

## Background

Central nervous system malignancies are the most common pediatric solid tumor type in North America [1]. They are the leading cause of death from disease in childhood and are the greatest source of cancer-derived morbidity in the USA, due to treatment side effects and resistance [2]. Pathologists commonly classify these tumors into glioma, ependymoma, and embryonal types [3]. Within these broad groups, there is substantial variability in histopathology and patient prognosis. For instance, gliomas encompass diagnoses such as glioblastoma, a high-grade cancer with low survival requiring chemotherapy, radiation, and surgery, while pilocytic astrocytoma, a benign entity, is cured by surgery alone. Due to the variation in underlying disease mechanism and impact on prognosis, risk stratification and classification are critical to treatment planning and goal setting.

Several studies have paved the way in using molecular markers to classify pediatric central nervous system (CNS) tumors. Through gene sequencing, diagnoses have been subdivided. *WNT1* and *SHH* mutations have been identified as subtype-specific genetic alterations in group 1 and 2 medulloblastomas with subsequent treatment and prognostic implications [4, 5] Likewise, ependymomas have been split into multiple subgroups based on *C11orf95-RELA* and *Yap1* fusions [6], while *BRAF-KIAA1549* fusion has been used to characterize pediatric low-grade gliomas [7]. These molecular markers have recently been incorporated into the WHO CNS tumor classification guidelines [3]. However, while genomic approaches have provided ways to better characterize pediatric CNS tumors, many lack obvious genetic drivers. Further, these genetic findings are not always associated with prognosis.

While genome-scale 5mC profiles have been well-described and used clinically in CNS tumors, there is very limited nucleotide-specific data for the other major cytosine modification in the brain, 5-hydroxymethylcytosine (5hmC). In mammals, TET proteins oxidize 5mC to 5hmC, which can be further transformed to 5-formylcytosine (5fC) and 5-carboxylcytosine (5caC) leading to methylation loss [8, 9]. Though initially thought to be an intermediate, 5hmC has since been shown to play a distinct role in regulating gene transcription and increasing chromatin accessibility [10–12]. Unlike methylcytosine, 5hmC concentrates in gene bodies and enhancers and is associated with increased gene expression [13–15]. 5hmC tends to be depleted in promoter CpG islands and is instead observed in the flanking CpG shores and shelves [14, 16].

5hmC modifications are most abundant in the brain, with levels tenfold higher than other tissues, though varying among regions of the brain [17]. In the cerebellum, 5hmC accounts for up to 42% of cytosine modifications [18]. Appropriate patterning of 5hmC is likely critical to normal neurodevelopment, and altered 5hmC has been associated with developmental neuropathologies such as Rett syndrome, autism, and schizophrenia [19–21]. 5hmC has been studied in adult brain tumors, such as glioblastoma, and has been shown to correlate with survival [13, 22, 23]. However, genome-wide nucleotide level studies in pediatric central nervous system tumors are lacking. Changes in 5hmC in tumors have been consistent across tissue types, with depletion of 5hmC in gene bodies and regulatory regions such as enhancers and transcription factor binding sites [24–28]. Loss of 5hmC tends to coincide with commonly mutated genes in cancers and is tissue-specific [10, 13, 29, 30]. 5hmC appears to play a unique role in cancer, such as localizing to sites of DNA damage, and promoters with 5hmC have been shown to be resistant to the hypermethylation that is common in cancers [28, 31]. To date, the role of 5hmC in pediatric CNS tumors has yet to be adequately measured and understood.

Epigenetic profiling to characterize CNS tumors in both research and clinical settings is increasingly applied. DNA methylation, the addition of a methyl group to cytosine, 5-methylcytosine (5mC), is one of the most common and best-studied epigenetic modifications. Alterations in the methylation status of specific cytosines have been used to classify CNS tumors in both adults and children to great effect—identifying changes that transcend somatic genetic alterations [32–36]. Capper et al. have even set up a molecular neuropathology webite where methylation array data can be uploaded to classify CNS tumors [36]. Tumor DNA methylation signatures have been used not only for diagnostic purposes but to stratify subgroups with a worse prognosis [37, 38]. However, current methods of measuring nucleotide level 5mC—including those for CNS tumor classification—rely on bisulfite conversion techniques which cannot distinguish between 5hmC and 5mC [39]. This confounds to what degree these classifications rely on 5hmC to diagnose or prognosticate tumor types and has not been clearly delineated.

Most studies of 5hmC only provide a genome-level summary measure of 5hmC without chromosome, gene region, or nucleotide-specific information [40, 41]. Due to the abundance of 5hmC in the brain and its observed alterations in tumorigenesis, we aimed to examine 5hmC in pediatric CNS tumors. We measured 5-methylcytosine and 5-hydroxymethylcytosine epigenome-wide at nucleotide resolution in pediatric glioma, ependymoma, and embryonal tumors as well as non-tumor tissue using tandem bisulfite and oxidative bisulfite sequencing followed by hybridization to the Illumina Methylation EPIC Array that interrogates over 860,000 CpG loci. Lastly, we used our raw data, processed with bisulfite treatment (including both 5mC and 5hmC) as well as oxidative bisulfite-treated data files (including only 5mC), as the input for several existing tumor classification systems to determine whether using 5mC-specific data could impact the diagnostic aptitude of these tools.

## Results

### Overview of study population

All cases included in this study (*n* = 27) received treatment at the Children’s Hospital at Dartmouth Hitchcock and the Norris Cotton Cancer Center between 1993 and 2009. The mean age of cases was 9.5 years, and the mean age of subjects providing non-tumor samples was 6 years. Tumor samples included thirteen gliomas, eight ependymomas, and six embryonal tumors. Additional details of subject demographic, tumor characteristics and diagnoses are provided in Table 1. Our cohort included both common and rare entities, including dysplastic gangliocytoma and spinal myxopapillary ependymoma. The most common glioma subtype was pilocytic astrocytoma, and medulloblastoma for embryonal tumors. The most diverse subtype was gliomas with a range of histopathologic diagnoses incluging desmoplastic ganglioglioma, dysembryoplastic neuropithelial tumors, and dysplastic gangliocytomas with limited sample sizes due to their respective rare prevalence (Table 1). All samples were *H3F3A (K27* and *G34*) and *TERT* promoter (C228T and C250T) wild-type, as determined by Sanger sequencing (Additional file 6: Table S1).

**Table 1:** Patient demographics and tumor characteristics

	Tumor (*n* = 27)	Non-Tumor (*n* = 3)
Age, mean (SD)	9.5 (5.5)	6 (5.6)
Range	1–18	0–11
*Sex n (%)*		
Female	12 (44)	1 (33)
Male	15 (56)	2 (67)
Tumor type,^a^ *n* (%)		
*Embryonal*	6 (22)	
Medulloblastoma	4	
Embryonal (NOS)	2	
*Glioma*	13 (48)	
Anaplastic ganglioglioma	1	
Desmoplastic ganglioglioma	2	
Dysembryoplastic neuroepithelial tumor	2	
Dysplastic gangliocytoma	1	
Glioblastoma	1	
Pilocytic astrocytoma	5	
Low-grade glioma	1	
*Ependymoma*	7 (30)	
Subependymoma	1	
Ependymoma	5	
Myxopapillary ependymoma	1	
*WHO grade n (%)*		
I	12 (44)	
II	5 (19)	
III	2 (7)	
IV	8 (30)	
*Sample location n (%)*		
Supratentorial	9 (33)	3 (100)
Subtentorial	17 (63)	
Spinal	1 (4)	
Median follow up (years)	14	
Mean time to recurrence (years)	1.5	

^a^Tumor type and grade were determined by a neuropathologist re-review of samples and distribution of tumor type was determined by prevalence at the institution

### Pediatric CNS tumors are globally depleted of 5hmC

To measure nucleotide-specific 5hmC and 5mC levels, we applied tandem bisulfite and oxidative bisulfite treatment to DNA from 27 fresh frozen pediatric CNS tumor samples and three non-tumor control tissues. Treated DNA was hybridized to the Illumina Human Methylation EPIC array. Following processing, CpG probes associated with SNPs and sex chromosomes were removed, and 743,461 CpG sites remained in the dataset.

**Fig. 1:**
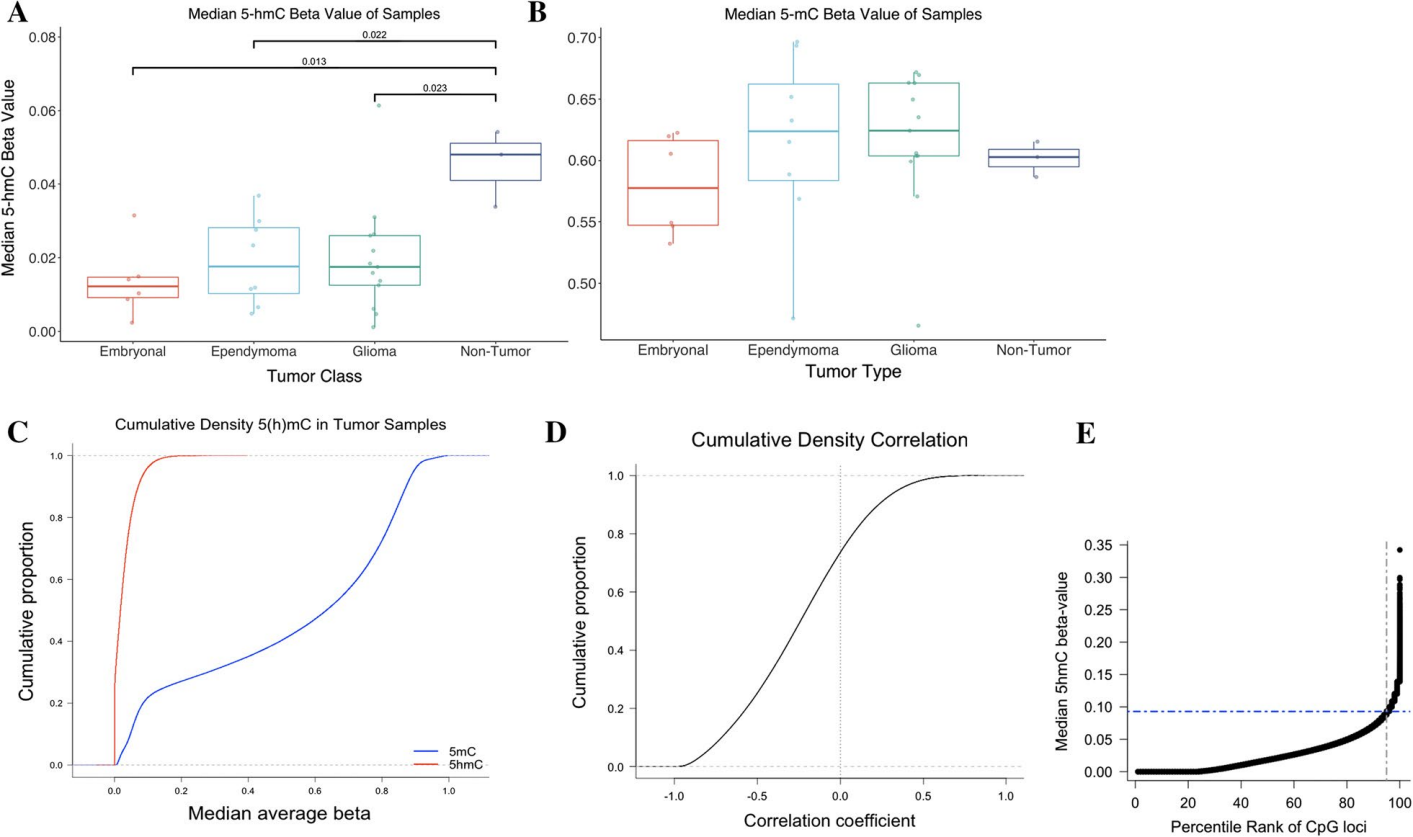
Distribution of 5-hmC in tumor samples and median beta values by tumor class and cytosine modifications in CNS tumors as compared to non-tumor brain tissue. **a** Consistent with studies in other tumor types, 5-hydroxymethylation levels are depleted compared to non-tumor samples; this depletion holds across tumor types. Two-tailed Welch’s *t* test comparing median values was performed and differences met statistical significance. **b** Total methylation levels do not differ significantly between tumor and non-tumor samples. **c** Examined the empirical cumulative distribution of median 5-hydroxymethylcytosine and 5-methylcytosine across 27 primary pediatric central nervous system tumors. While 5mC demonstrates a bimodal distribution, 5hmC is far more sparsely distributed. **d** Cumulative proportions of Spearman correlation coefficients calculated for each CpG across all tumors. **e** Ordered distribution of CpG-specific median 5hmC values across the EPIC array in tumor samples. The *x* axis represents percentile rank of CpG by median beta value. We isolated CpGs with medians greater than the 95th percentile and called these high 5hmC CpGs. This corresponded to 37, 173 loci with a median beta value greater than 0.09. We based subsequent analyses on these high 5hmC CpGs

To first compare our findings with prior work where 5hmC measures only provide a single epigenome-wide summary value, we calculated median 5hmC levels using the 743,461 CpG sites in our dataset. Median 5hmC levels were significantly lower in tumors (gliomas 1.75%, ependymomas 1.76%, and embryonal tumors 1.22%), compared to non-tumor tissues (4.81%) (Fig. 1a). Median levels of tumor 5mC (glioma 62.5%, ependymoma 62.4%, embryonal 57.7%, non-tumor 60.3%), did not differ from controls (Fig. 1b).

Cumulative density plots for median 5hmC and 5mC across all tumor samples demonstrated very low 5hmC levels at the majority of CpG sites in contrast to 5mC levels, which were more evenly distributed (Fig. 1c). We observed a negative correlation between 5hmC and 5mC for approximately 80% of CpGs in tumor samples as indicated by the distribution of Spearman’s correlation coefficients (Fig. 1d). CpG-specific median beta values were calculated across all tumor samples and ordered by increasing value to identify the distribution of 5hmC level and loci with appreciable 5-hydroxymethylation (Fig. 1e).

**Fig. 2:**
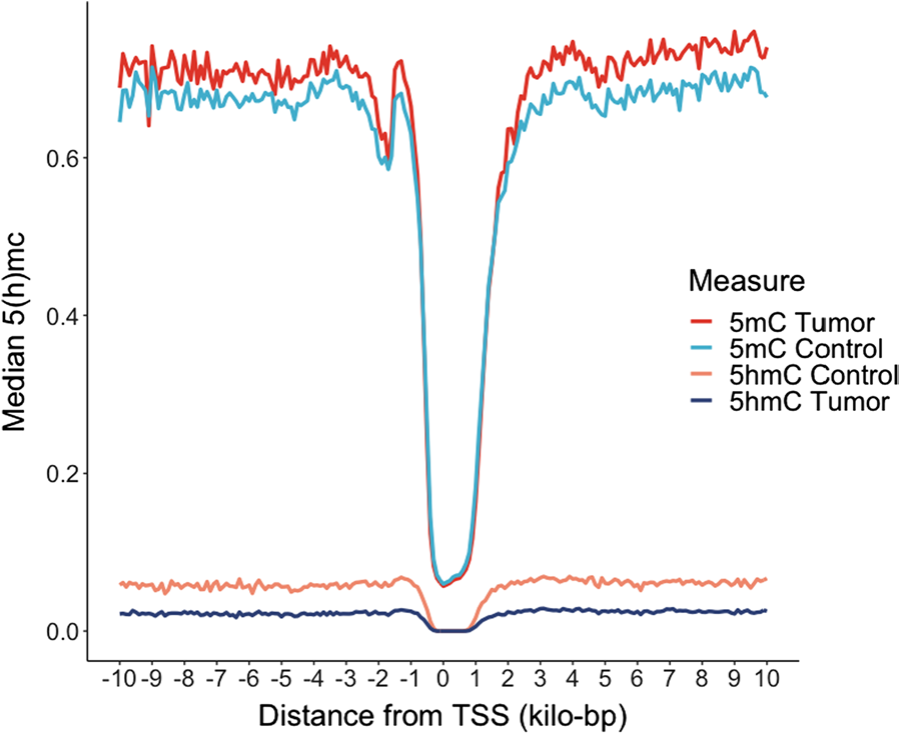
5hmC levels in relation to transcription start sites. Median 5hmC and 5mC within ± 10 kilo base pairs from the nearest gene transcription start site (TSS) for both tumors (*n* = 27) and non-tumors (*n* = 3). We observed that there were minimal differences between tumors and non-tumors near the transcription start sites and both cytosine modifications were depleted. However, within less than 1000 base pairs of the TSS, tumors were hypohydroxymethylated and hypermethylated as compared to controls

The difference in the levels of 5hmC and 5mC between tumors and controls depends on the distance from the transcription start site (TSS). Immediately surrounding the TSS, there was no discernible difference between tumors and non-tumor tissues, and levels of both cytosine modifications drop to near zero. At approximately 300 base pairs in the 5′ and 700 base pairs 3′ direction, tumors demonstrate hypohydroxymethylation and hypermethylation (Fig. 2). Compared with non-tumor control tissue, we observed consistently lower levels of 5hmC within each tumor subtype (Additional file 6: Fig. S1a). Despite lower median 5hmC near TSSs, a substantial proportion of high 5hmC CpGs are found within 5 kb of the TSS, with 14% of loci within 2000 base pairs upstream of the start site (Additional file 6: Fig. S1b).

We decided to focus on the top 5% of measured loci with the highest median 5hmC beta values, which we refer to as the "high 5hmC CpGs," for downstream investigation. This subset consisted of 37,173 loci with a median beta value greater than 0.09 (Fig. 1d). 5mC levels at these sites had similar relationships between tumors and non-tumors as at the genome wide level with tumor median levels significantly higher than non-tumor (Additional file 6: Fig. S2).

### Genomic enrichment of 5hmC in regulatory regions of the genome

**Fig. 3:**
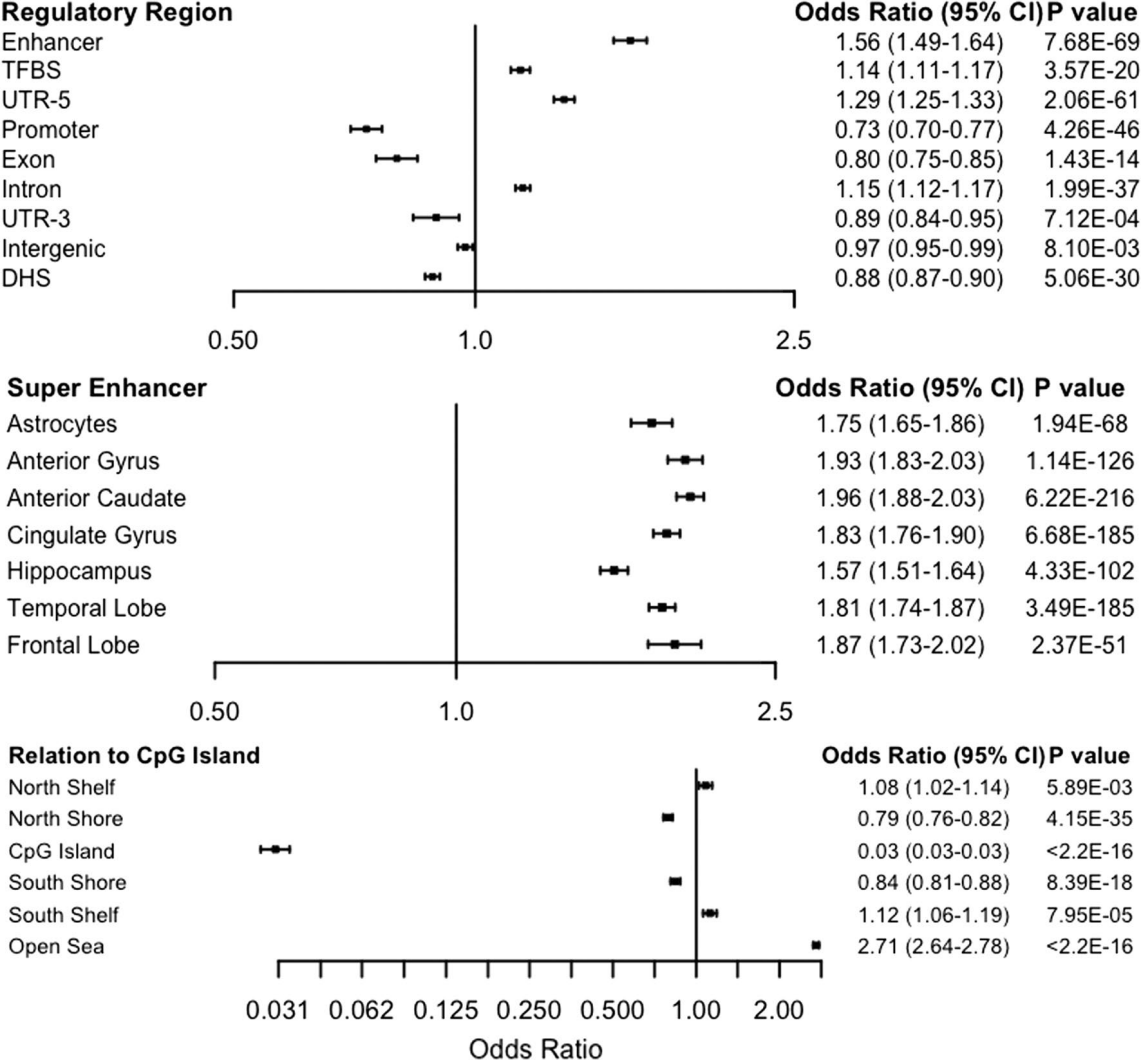
Enrichment of high 5hmC nucleotides in transcriptional regulators. High 5hmC loci are modestly enriched in enhancers, transcription factor binding sites, and 5′ untranslated regions. Conversely, they are depleted in promoters. Here, the top 37,173 CpGs are compared to all the sites included in the EPIC methylation array. Compared to the 850k universe, these sites are significantly depleted in CpG islands and enriched in open sea regions. These CpGs are enriched in super enhancers suggesting a role in determining cellular identity. The x-axis for relation to CpG islands represents the log odds ratio

We then investigated the relationship between genomic context and CpGs with high 5hmC, specifically investigating CpG islands and other genomic transcriptional regulatory elements (Fig. 3). Tumor CpG islands are significantly depleted of high 5hmC CpGs (OR 0.03, 95% CI 0.03–0.03, *P* < 2.2E−16). Out of the 37,173 loci designated as high 5hmC, only 281 were identified as residing in the CpG islands. CpG depleted open sea regions are significantly enriched for high 5hmC CpGs (OR 2.71, 95% CI 2.64–2.78, *P* < 2.2E−16). In fact, 77% of high 5hmC loci were in open sea regions (Additional file 6: Table S2). High 5hmC sites predominantly localize to genomic regions associated with transcriptional regulation such as enhancers (OR 1.56, 95% CI 1.49–1.64, *P* = 7.68E−69), transcription factor binding sites (OR 1.14, 95% CI 1.11–1.17, *P* = 3.57E−20), and 5′ untranslated regions (UTR-5) (OR 1.29, 95% CI 1.25–1.33, *P* = 2.06E−61).

Super-enhancers have been shown to be critical to cell identity, and aberrant methylation in these regions has been implicated in the development of human cancer [42]. To test the relation of super-enhancer with high 5hmC loci, we accessed super-enhancer coordinates defined in multiple brain-derived cell lines from the dbSuper database [43] and performed the same enrichment analysis as above. We identified significant enrichment of high 5hmC in annotated super-enhancers, including astrocyte lines (OR 1.75, 95% CI 1.65–1.86, *P* = 1.94E−68) and anterior caudate lines (OR 1.93, 95% CI 1.88–2.03, *P* = 6.22E−216, Fig. 3).

Next, we investigated the enrichment of high 5hmC loci at the gene level. We wanted to identify genes that had large clusters of high 5hmC loci. We first identified genes with at least ten high 5hmC CpGs (*n* = 486 genes). We defined the gene-level high 5hmC sites as a proportion of measured CpGs that were high 5hmC to account for different gene lengths, number of CpGs measured on the array, and number of high 5hmC CpGs. The range of high 5hmC sites as a proportion of those measured ranged from 1.4 to 52.6% and the mean proportion of hydroxymethylated CpGs among the 486 genes was 17%. Genes with the highest proportion of their CpGs designated as “high 5hmC” were *SHOC2* (53%), *ZRANB1* (48%), *MBNL1* (41%), *ZMIZ1-AS1* (35%), *DICER1* (35%), and *GNAQ* (35%) (Additional file 2). This list also included, but was not exclusive to, genes pertaining to RNA interference pathways such as *DICER1* and Argonaute gene (*AGO2*) [44–46] and neurodevelopment (*GNAQ*, *FOXO3*) [47, 48]. Using the genomic coordinates of the list of genes enriched for high 5hmC loci as the input, the Genomic Regions Enrichment of Annotations Tool (GREAT) analysis revealed biological pathways including regulation RNA interference pathways and RISC complex formation, as well as cell signaling pathways, and neuronal signaling and development. High 5hmC pathways also included those associated with craniofacial and neurodevelopmental pathologies, including autism, hyperactivity, and slanted palpebral fissures (Additional file 2).

### Locus-specific differentially hydroxymethylated regions

**Fig. 4:**
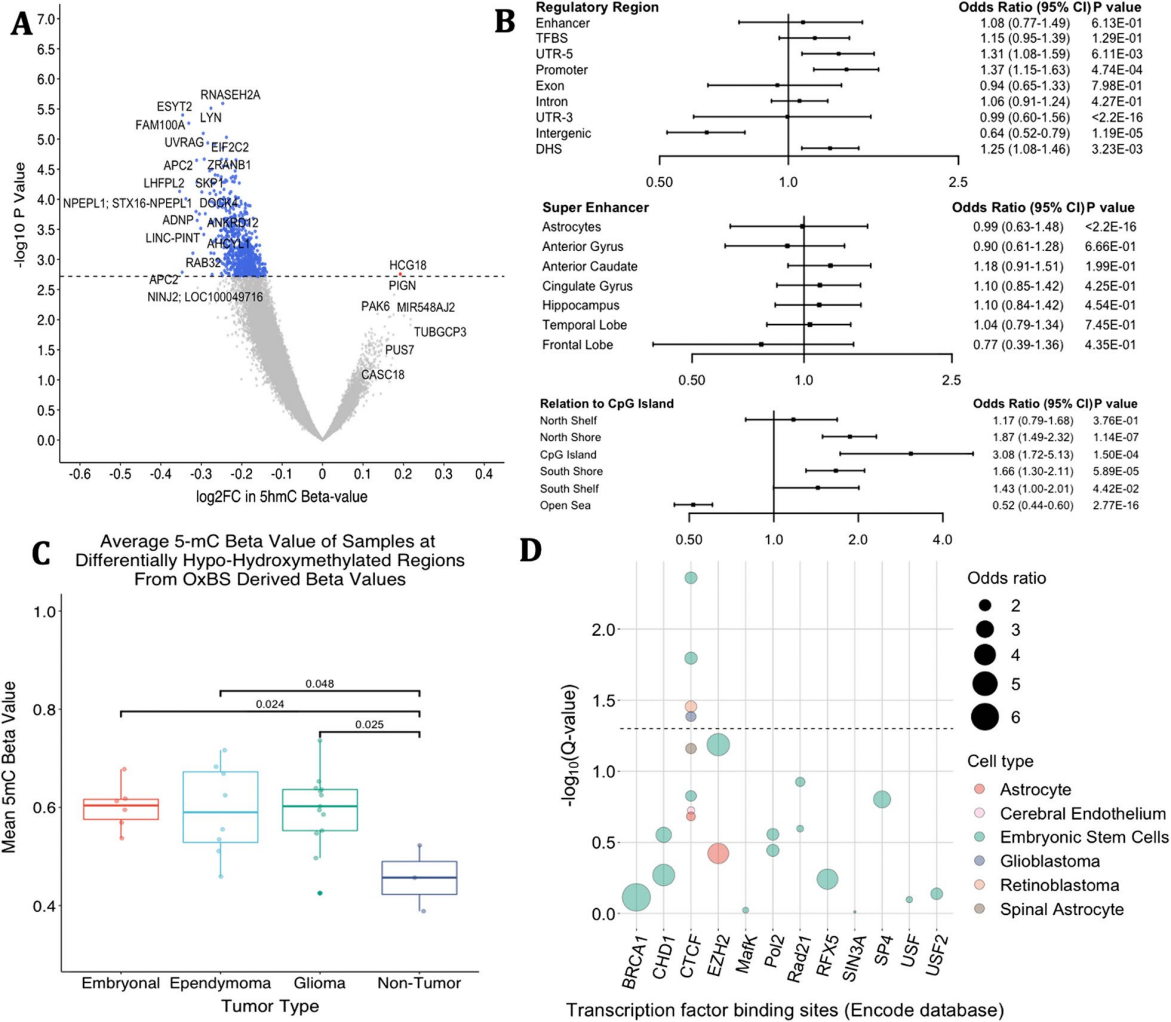
Localization of differentially hydroxymethylated regions. **a** EWAS results of the high 5hmC CpGs (*n* = 37,173) comparing tumor and non-tumor tissue from an age and sex adjusted linear model (limma). Volcano plot of differentially hydroxymethylated CpGs, plotting the difference of log2 fold change in beta value and the respective negative log10 of the unadjusted *P* value. Blue points represent 726 differentially hypohydroxymethylated regions (DHMR) with an adjusted *P* value (FDR) < 0.1. **b** Forest plot demonstrates enrichment of differentially hydroxymethylated regions within the high 5hmC loci. **c** Oxidative bisulfite derived mean 5mC beta values at these DHMRs stratified by tumor type demonstrates significant hypermethylation at these loci in tumors as compared to controls. However, bisulfite treatment alone only cannot distinguish between 5mC and 5hmC and demonstrated no change or hypomethylation. **d** We leverage the locus overlap analysis package (LOLA) to determine the significance of overlap of hypohydroxymethylated sites with binding sites of specific transcription factors profiled by ENCODE. The top 12 enriched transcription factors discovered by LOLA analysis are represented above. TFs are plotted on the *x* axis and the *y* axis represents the -log10 *q* value (corrected for multiple hypothesis testing). The size of the bubbles represents the odds ratio and the color represents the cell line. The dotted line represents a *q* value of 0.05. CTCF is the only transcription factor that meets statistical significance

We tested the high 5hmC sites for differential hydroxymethylation between tumor and non-tumors (*n* = 37,173), adjusting for age and sex, in a linear model. Tumors were hypohydroxymethylated, with 84% of the high 5hmC loci demonstrating reduced hydroxymethylation, and we observed 726 loci with significantly hypohydroxymethylation in tumors (FDR < 0.1, Fig. 4a). Submitting the 726 differentially hypohydroxymethylated regions (DHMRs) to GREAT analysis resulted in overlap with steroid response pathways (4 of the 20 gene ontology biological processes), RNA stability, as well as WNT signaling and beta-catenin binding (Additional file 3). Furthermore, in contrast to the overall genomic context patterning of high 5hmC CpGs, tumor DHMRs were significantly enriched in CpG islands and shore regions while depleted in open sea regions (Fig. 4b). The majority of CpGs, 63%, were found in open sea regions (Additional file 6: Table S3). Differentially hydroxymethylated regions (DHMRs) in CpG islands were associated with 10 genes which included Wnt pathway regulator *APC2,* histone H3 demethylase *KDM2A*, and orphan G protein-coupled receptor GPRC5B critical for neurodevelopment (Additional file 3) [49].

To determine whether particular transcription factors are associated with DHMRs, we tested for enrichment in binding sites from transcription factor experiments (ENCODE) using the locus overlap analysis (LOLA) software in CNS and embryonic stem cell lines. We identified enrichment for DHMRs at CTCF binding sites, a transcription factor linked to alternative splicing by regulating RNA polymerase II and TET [50] (Fig. 4d). Using LOLA to interrogate sites associated with histone modifications generated by the NIH Roadmap Epigenomics Project, we found that DHMRs were significantly associated both with chromatin signatures for transcriptional activation (H3K4me3) and priming (H3K4me1) (Additional file 6: Fig. S3). Lastly, we investigated total 5mC levels among DHMRs. All tumor subtypes were hypermethylated at these sites as compared to non-tumors (Fig. 4c). This pattern of hypohydroxymethylation and hypermethylation was only captured in tandem OxBS derived data and was lost in BS only derived data with the latter demonstrating a relative hypomethylation in tumors (Additional file 6: Fig. S4). In tumors, these sites also demonstrated higher levels of 5mC compared to average 5mC levels in the rest of the genome (Additional file 6: Fig. S5).

### Subtype to non-tumor comparisons

**Fig. 5:**
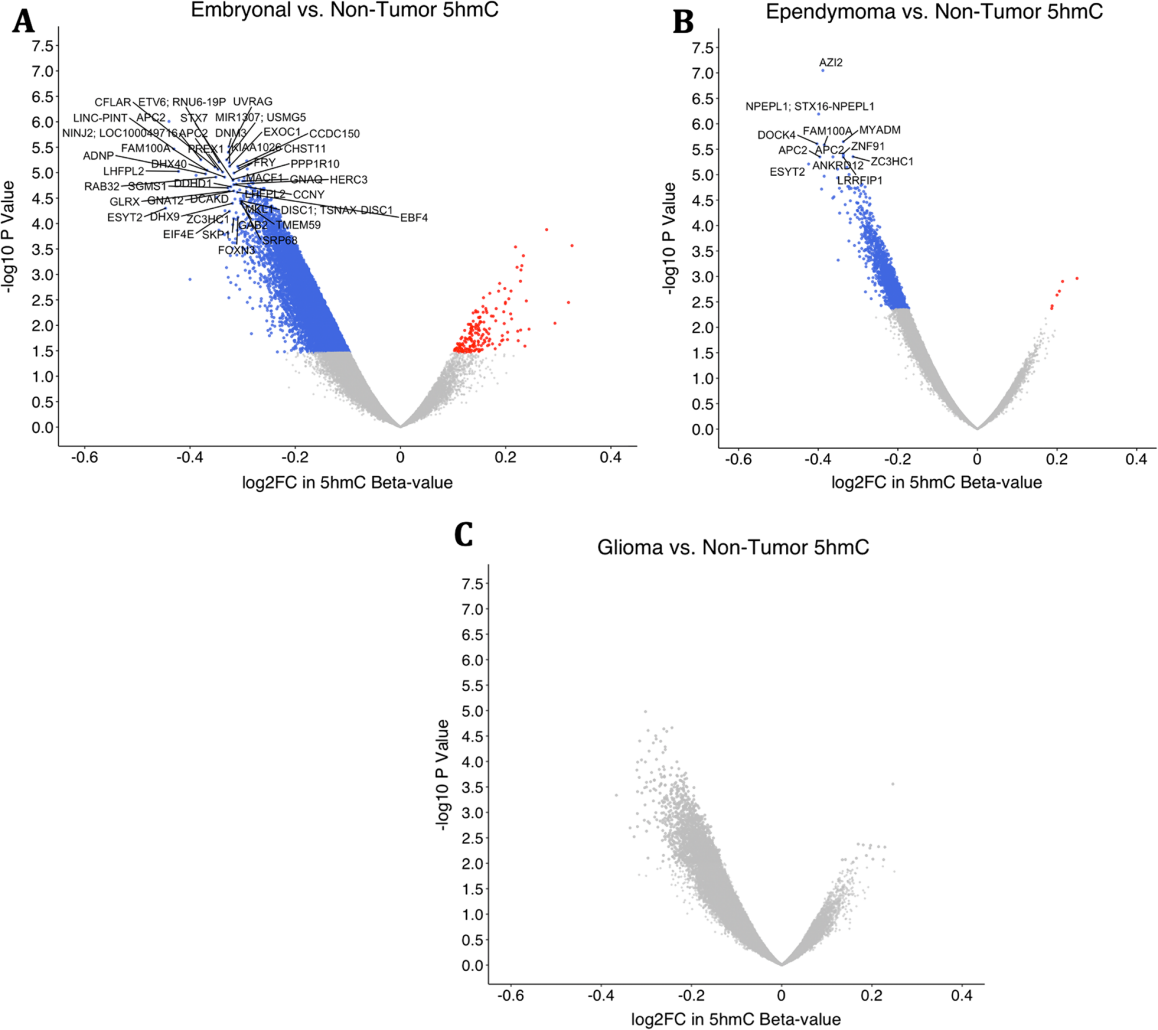
Results from linear models comparing CpG hydroxymethylation in each tumor subtype to non-tumors. Blue represents loss of 5hmC and red gain of 5hmC by tumor as compared to controls. **a** Volcano plot of differentially hydroxymethylated regions in a comparison between embryonal and control samples. Colored points represent CpGs that are differentially hydroxymethylated with an adjusted *P* value (FDR) less than 0.1. 15,072 CpGs of the 37,173 high 5hmC CpGs were significantly differentially hydroxymethylated with an FDR less than 0.1. **b** Volcano plot comparing ependymomas to non-tumors. Colored points represent an FDR less than 0.1. **c** Comparison of gliomas to controls. No points reached statistical significance

To further explore high 5-hmC sites (*n* = 37,173) in tumor and non-tumor tissues, we stratified by tumor type (embryonal, ependymoma, glioma) and compared to non-tumor tissues adjusting for age and sex. Embryonal tumors had the most extensive differential hypohydroxymethylation with 12,593 loci (40%) losing 5hmC (FDR < 0.1, (Fig. 5a). We subset these loci to CpGs with an FDR < 0.02 (*n* = 1832) and performed a GREAT analysis. The previously observed response to steroid pathways was maintained, and pathways related to mTOR regulation emerged (Additional file 4). The extent of differential hydroxymethylation in ependymomas compared with non-tumor tissues was lower than that of embryonal tumors (*n* = 1630 DHMR, FDR < 0.1, Fig. 5b). We did not observe significant differential hydroxymethylation among gliomas compared with non-tumor samples’ DHMRs (Fig. 5c). Though gliomas did not appear to be significantly differentially hydroxymethylated as compared to controls, we questioned whether this was due to the inherent bias of subsetting our analysis to the high 5hmC loci. Therefore, we ran the linear model on all loci in the array and found 25, 147 differentially hydroxymethylated CpGs (Additional file 6: Fig. S6). We repeated GREAT analysis for ependymomas with loci with an FDR < 0.1 (*n* = 1630) and gliomas with an FDR < 0.2 (*n* = 637) (Additional file 4). Response to corticosteroid pathways was consistently present across all tumor types.

### 5hmC and survival

**Fig. 6:**
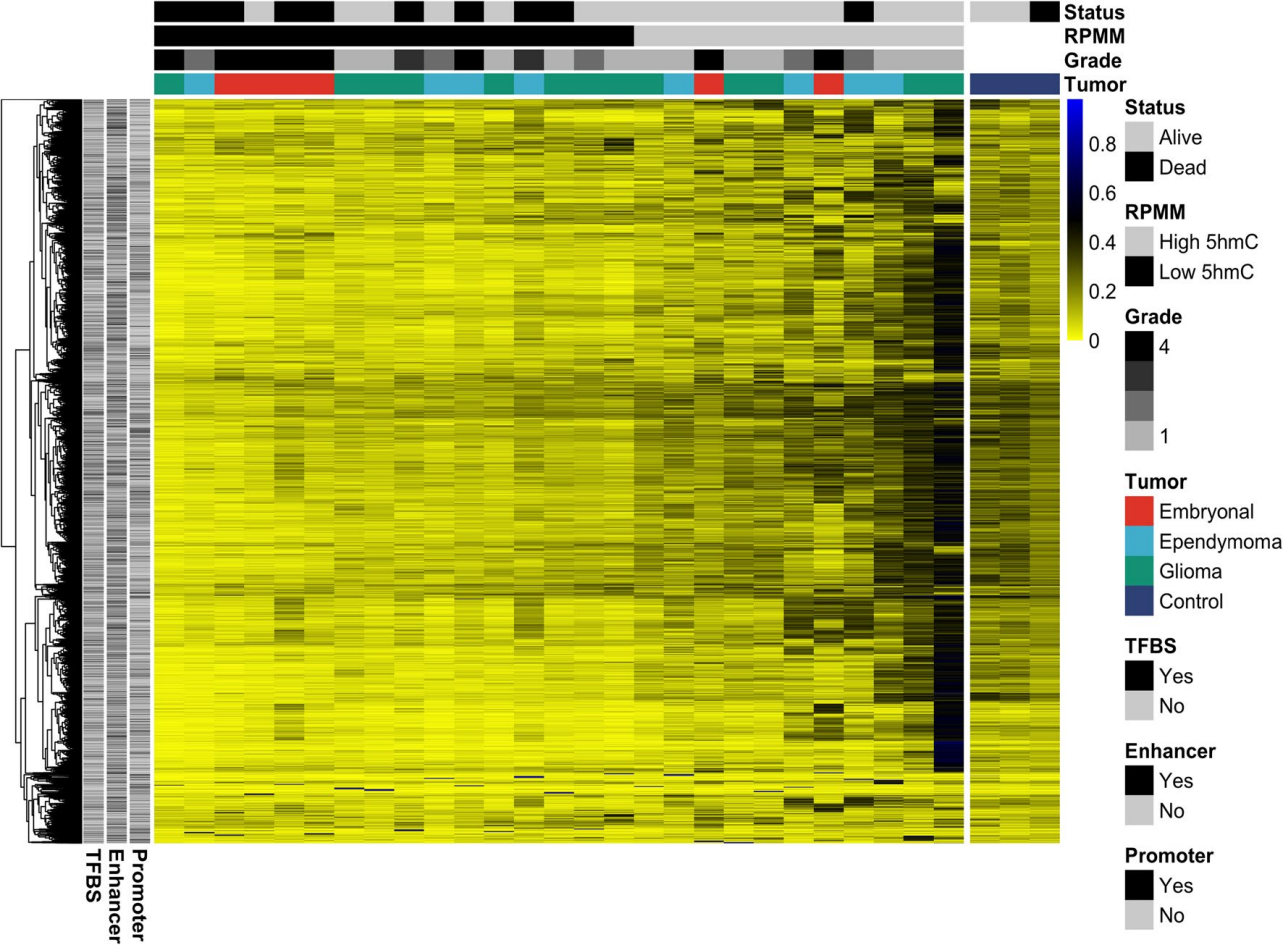
High and low 5hmC clusters and their relation to survival and recurrence. Recursively partitioned mixture model (RPMM) of tumor samples of the top 10,000 most variable CpGs in tumor samples. In the heatmap, each column represents an individual sample and each row is a CpG. Two clusters emerged that correlated with average 5hmC and were so labeled high 5hmC (gray) and low 5hmC (black). Patient status as alive or dead is denoted under status and tracks with RPMM cluster as does tumor grade

Prior publications identified an association between decreased total 5hmC and survival in CNS tumors with mixed results [13, 40]. Here, we used a model-based clustering method, recursively partitioned mixture model (RPMM) [51], to discern profiles of 5hmC and investigate the association of profiles with survival. Applying RPMM to the top 10,000 most variable sites in tumor samples yielded two classes of tumors, which correlated with mean 5hmC levels (*P* = 2.735E−05, Kruskal–Wallis rank-sum test, *df* = 1), and we then defined the two classes as high 5hmC and low 5hmC. A Chi-squared test evaluating the relationship between RPMM clusters and grade did not reach our threshold for statistical significance with a *P* value of 0.099. Similarly, we did not have sufficient evidence to conclude an association of RPMM cluster with tumor type (Embryonal OR 0.68, CI 0.05–6.03, *P* > 0.99, Ependymoma OR 1.68, CI 0.23–12.42, *P* = 0.67, Glioma OR 0.84, CI 0.14–4.98, *P* > 0.99) or subject age (Kruskal–Wallis rank-sum test *P* = 0.26, *df* = 1). With the exception of the glioblastoma sample which was excluded from our survival analysis due to lack of follow up and one anaplastic ganglioglioma, all gliomas were low grade. All embryonal tumors in our study were grade 4 and only 2 of 8 ependymomas were high grade. Despite this, there was relatively equal distribution of tumor types in high and low 5hmC clusters. The low 5hmC cluster was composed of 25% embryonal tumors, 25% ependymoma, 50% gliomas while the high 5hmC class had 18% embryonal, 36% ependymoma, 45% gliomas (Fig. 6). Then, we fit two independent multivariable Cox proportional hazards models for survival and recurrence adjusting for patient sex and tumor type, for the outcomes of death and recurrence. The low 5hmC cluster was associated with an increased hazard of death (HR 6.47, 95% CI 0.79–53.2, *P* = 0.08, concordance index = 0.84, Cox proportional hazards regression) and recurrence (HR 4.83, 95% CI 0.95–24.6, *P* = 0.06, concordance index = 0.79) (Table 2). Applying the model but adjusting for grade demonstrated similar trends of improved prognosis for patients with high 5hmC tumors, but were underpowered (Additional file 6: Table S4 and Fig. S7).

**Table 2:** Cox proportional hazard models of survival by RPMM 5hmC cluster membership

A			B		
Cox proportional hazard ratios for survival^a^			Cox proportional hazard ratios for recurrence^b^		
Variable	HR (95% CI)	*P* value	Variable	HR (95% CI)	*P* value
Age	0.97 (0.83–1.1)	0.70	Age	0.93 (0.80–1.1)	0.30
*Sex*			*Sex*		
Female	1.0 (referent)		Female	1.0 (referent)	
Male	0.73 (0.12–4.3)	0.73	Male	0.70 (0.20–2.4)	0.58
*Tumor type*			*Tumor type*		
Glioma	1.0 (referent)		Glioma	1.0 (referent)	
Ependymoma	3.93 (0.54–28.6)	0.18	Ependymoma	6.63(1.21 -36.3)	0.03
Embryonal	4.95 (0.63 -38.9)	0.13	Embryonal	4.27 (0.76 -24.1)	0.10
*RPMM cluster*			*RPMM cluster*		
High 5hmC cluster	1.0 (referent)		High 5hmC cluster	1.0 (referent)	
Low 5hmC cluster	6.47 (0.79–53.2)	0.08	Low 5hmC cluster	4.83 (0.95–24.6)	0.06

^a^Multivariate Cox proportional hazard ratios and confidence interval for survival based on RPMM cluster membership adjusted for age, sex, and tumor type demonstrated an increased hazard of death for the low 5hmC RPMM cluster (HR 6.47, 95% CI 0.79–53.2, *P* = 0.08)

^b^Multivariate cox proportional hazard ratio for recurrence based on RPMM cluster membership adjusting for age, sex, and tumor type demonstrated an increased hazard of recurrence for the low 5hmC cluster (HR 4.83, 95% CI 0.95–24.6, *P* = 0.06)

In order to compare our results to more common methods of correlating 5hmC with survival, we constructed an index representing the total 5hmC content. The index is defined by averaging beta-values of all CpGs (*n* = 743,461) and assigning a sample to a “High Total 5hmC” group if the mean was greater than the 50th percentile (i.e., the sample mean 5hmC level was greater than 0.035). The results were consistent with the RPMM method described above, with the low 5hmC group having poorer survival (log-rank *P* = 0.08, concordance index 0.75) (Additional file 6: Fig. S8). In a multivariable Cox proportional hazards model adjusted for sex and tumor type, the low total 5hmC group was associated with increased risk of recurrence though this did not reach statistical significance (HR 3.6, CI 95% 0.9–14.5, *P* = 0.07) and death (HR 3.4, CI 95% 0.69–16.5, *P* = 0.14).

Applying principal component analysis (PCA) of this subset of CpGs, revealed discernible clusters that overlapped substantially with the different tumor types (Additional file 6: Fig. S9a). PCA also showed an overlap of clusters with survival (Additional file 6: Fig. S9b). Using methylation beta values at these loci also yielded discernible cluster separation by survival emphasizing the potential clinical relevance of our CpGs (Additional file 6: Fig. S9c).

### 5hmC and methylation-based classification systems

We next assessed the robustness of epigenetic CNS tumor classification methods to array data that is 5mC-specific. Molecular neuropathology accepts bisulfite-treated IDAT files from CNS tumors and offers a list of diagnosis with best fit. We submitted IDAT files from both bisulfite and oxidative bisulfite-treated samples were submitted to this methylation-based CNS tumor classification system [36]. Despite high-quality data from fresh frozen specimens provided from the bisulfite-treated IDAT files, the molecularneuropathology.org tool could not classify 26% (7/27) of our tumors labeling them “Not Defined” or “Control tissue.” Undefined tumors included those with histopathologic diagnoses both common such as medulloblastoma and subependymoma as well as rare such as dysplastic gangliocytoma, desmoplastic and anaplastic ganglioglioma. Of particular interest, when the OxBS-treated IDAT files, representing 5mC-specific signal, from the same tumors were submitted, 26% (7/27) of the samples switched diagnoses (Table 3), suggesting that hydroxymethylated CpGs are included in the Capper et al. random-forest-based classification system. In addition, we used a pediatric focused methylation-based classification tool called MethPed [34, 35] to investigate 5mC-specific signal in tumor classification. While this tool only left the dysplastic ganglioglioma as undefined, it over diagnosed our samples as glioblastomas. Many of the samples that the Capper classification tool left undefined, MethPed predicted to be glioblastomas. The MethPed classification of OxBS derived 5mC-specific data resulted in 24/27 (89%) of tumors switching diagnoses. Further, when we tested the Capper Classification system on 5mC-specific adult glioblastoma data from our prior work on hydroxymethylation [13] 73% (22/30) of tumors changed diagnoses, suggesting that 5hmC plays an even greater role in adult glioblastoma classification than pediatric tumors. Most tumors were reclassified from IDH wildtype glioblastoma to “control tissue, inflammatory tumor microenvironment.” To assess whether tumor purity affected the diagnosis of the classification systems, we derived purity estimates with the Infinium Purify [52] method. There was no association between class switching and tumor purity for either the MethPed (ANOVA, *P* = 0.7) or Capper classification systems (ANOVA, *P* = 0.361).

**Table 3:** Methylation-based classification systems and tumor type predictions

Capper methylation-based classification tool	BS (*n*)	OxBS (*n*)	MethPed methylation-based classification tool	BS (*n*)	OxBS (*n*)
*Glioma*	11	9	*Glioma*	15	18
Anaplastic pleomorphic xanthoastrocytoma	1	1	Pilocytic astrocytoma	7	11
CNS high-grade neuroepithelial tumor with MN1 alteration	1	1	Glioblastoma	8	7
Pilocytic astrocytoma	6	6			
Glioma, IDH mutant	1	1			
Low-grade glioma, dysembryoplastic neuroepithelial tumor	2				
*Ependymoma*	5	4	*Ependymoma*	5	5
Ependymoma, myxopapillary	1	1			
Ependymoma, posterior fossa group A	3	2			
Ependymoma, RELA fusion	1	1			
*Embryonal*	4	4	*Embryonal*	6	4
Medulloblastoma group 3 and 4	4	4	Medulloblastoma SHH	1	1
			Medulloblastoma group 3 and 4	4	3
			Embryonal tumor with multilayered rosettes	1	
*Undefined*	7	10	*Undefined*	1	0
Not defined	6	6			
Control tissue	1				
Plexus tumor		3			
CNS neuroblastoma with FOXR2 activation		1			

^a^Sample IDAT files from both BS and OxBS-treated tissues were submitted to methylation-based CNS tumor classification systems: molecular neuropathology and MethPed. The table above demonstrates how classifications switched in pediatric tumors based on the classification system and the files submitted

**Fig. 7:**
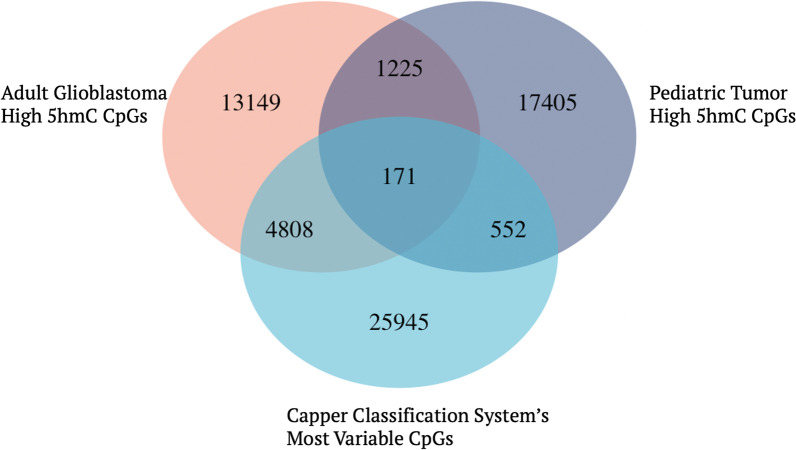
Venn diagram of shared CpGs in adult and pediatric tumor high 5hmC loci and methylation-based classification system’s most variable loci. The overlap of high 5hmC sites identified in pediatric tumors and adult glioblastomas with the most variable CpGs in the Capper data set with high 5hmC loci in pediatric and adult CNS tumors. There was considerably greater overlap between adult glioblastoma’s high 5hmC loci and the Capper data sets most variable CpGs (18%) than with the pediatric tumors’ high 5hmC CpGs (2%)

To compare overlap of our high 5hmC with CpGs used in the Capper unsupervised analysis, we explored the overlap between the most variable CpGs in the Capper dataset and the high 5hmC CpGs in our pediatric cohort and adult glioblastomas. We isolated 31,476 with a S.D > 0.228 to capture an approximation of the 32,000 most variably methylated probes mentioned in the original paper’s methods. We then reprocessed the pediatric and adult samples to identify the top 5% most hydroxymethylated CpGs in both groups. 723 of the 19,353 (3.7%) pediatric high 5hmC CpGs overlapped with the Capper set. Consistent with the large proportion of tumors that switched classes for adult glioblastomas, 4979 of the 19,353 (26%) high 5hmC CpGs from that data set overlapped with the Capper classification CpG set (Fig. 7). With regard to the MethPed probes, the 900 CpGs used by their classification system were published and we found that 45 of the 900 probes overlapped with the high 5hmC CpGs in the pediatric study.

## Discussion

Childhood nervous system tumors have a relatively low incidence of mutations. Common variations tend to be related to epigenetic mechanisms such as chromatin remodeling [53–55]. DNA methylation has been explored as a potential driver, and our results delineate the unique role of 5hmC from 5mC in CNS tumor pathology. We demonstrate that 5hmC is lost in tumors in a locus-specific pattern and that these sites are hypermethylated. This mirrors findings in adult glioblastomas, which undergo a similar exchange of 5hmC for 5mC [23]. CpG islands that were specifically affected by this pattern include *APC2*, a regulator of WNT signaling pathway, and *KDM2A,* a histone demethylase.

CNS tumor classification systems such as MethPed and Molecular Neuropathology rely on bisulfite-treated data that do not distinguish between 5hmC and 5mC. Our study suggests for the first time that 5hmC affects methylation-based CNS tumor classification systems as OxBS files which contain 5mC levels changed diagnoses. Oxidative bisulfite treatment enabled us to resolve this relationship, whereas bisulfite only treated samples—which measure the sum of 5hmC and 5mC—demonstrated hypomethylation in tumors as compared to controls. This was independent of tumor purity and more pronounced in adult glioblastomas than pediatric tumors. We postulate that given the high rate of change in classification with the OxBS data that 5hmC does play a role in the classification of these tumors but that the CpGs used to assign pediatric CNS tumor diagnosis are not necessarily those with the highest 5hmC levels. This is in contrast with adult glioblastomas which not only demonstrated a higher rate of change but also had 26% of the high 5hmC loci in the most variable loci of the Capper dataset.

It appears that loci that are critical to tumor classification are not necessarily the most hydroxymethylated due to little overlap of loci that are used in these classification systems with our list of high 5hmC CpGs. Our study as well as previously conducted projects have focused on the most hydroxymethylated loci as these tend to have the clearest signals and are less likely to be attributable to noise alone. However, our findings motivate further examining loci with median levels of 5hmC. Our results reinforce the importance of a more nuanced approach to the study of cytosine modifications in CNS tumors, and offer new opportunities to refine classification approaches that benefit clinicians and patients [36].

Though 5-hydroxymethylation in pediatric brain tumors has been investigated, studies to date have not been on a genome-scale at a locus resolution. An association between 5hmC and anaplasia was established using immunohistochemistry (IHC) to examine 5hmC in all WHO classifications of brain tumors [56]. Our study adds to existing literature that 5hmC accumulates in super-enhancers and suggests a link between a loss of 5hmC and anaplasia [56]. Recently, Wu et al. showed high 5hmC levels were associated with worse survival in pediatric posterior fossa ependymomas [40]. However, as this study was conducted using IHC, it remains unclear where in the genome this hyperhydroxymethylation was taking place. In line with previous studies, we observed that the loss of 5hmC in tumors was associated with shorter overall survival and time to recurrence [13, 22]. A nucleotide level analysis allowed us to identify enrichment of high 5hmC loci in genes critical to normal craniofacial and neurodevelopment further strengthening the link between these tumors and developmental neurobiology [57]. We also found that differentially hypohydroxymethylated CpGs are enriched in molecular pathways that are frequently connected to childhood brain tumors, particularly those related to *WNT* signaling and beta-catenin binding, implicating such genes as *APC2*, *WNT4*, *WNT11*, *WNT5B*, *SHH*, and *BCL9*. 5hmC loss in *WNT* has been associated with other tumors such as melanoma and colorectal cancer [26, 30].

The tendency of 5hmC to accumulate at 5′ splicing sites in the exon–intron boundary has been suggested as a link between the epigenetic marker and alternative splicing [58]. We found that not only did our high 5hmC sites localize to 5′ untranslated regions in concordance with previous work but that these loci were enriched in genes implicated in posttranslational regulation of gene expression such as the *DICER1*, *AGO2*, and *EIF2C2*. The association between 5hmC and CTCF, a methylation-sensitive transcription factor linked to alternative splicing and RNA polymerase II regulation, has also been reported in embryonal cells [12, 59]. Increased 5hmC levels have been tied to reduced binding of nucleosomes to DNA and reduced CTCF attachment [60]. 5hmC has been shown to oscillate at 150 nucleotides, the length of nucleosome wound DNA, and 5hmC has been suggested as a linker binding CTCF to DNA [61].

Due to the extreme rarity of pediatric brain tumors, our study is limited by the sample size. Additionally, our study consists primarily of medulloblastomas, pilocytic astrocytomas and ependymomas based on the prevalence of these tumors therefore generalizable largely to these subtypes. Larger studies would need to be done in order to capture the true effect of 5hmC on pediatric CNS tumors. In the future, a larger multi-institutional study is warranted to narrow the confidence interval for 5hmC association with patient outcomes and increase power for differential hydroxymethylation analysis in tumor subgroups. We did not observe significant differential hydroxymethylation among gliomas compared with non-tumor samples, which is not unlikely a reflection of the heterogeneity of this tumor class. These tumors were diagnosed prior to the WHO 2016 Classification of CNS tumors [3] with the updated molecular studies that it entailed, and it is possible that some of these tumors would be diagnosed differently if the most recent criteria were applied. However, histopathological re-review was conducted for all samples. Though potential variation in the proportion of non-tumor tissue content may have contributed to more proximal clustering with non-tumor samples and higher total 5hmC levels, the observed association of 5hmC with survival and recurrence was robust to this potential variation. Including multiple types of pediatric central nervous system tumors, both rare and common, and comparing tumor tissue to control samples enhances the generalizability of our findings. In the future, 5hmC measures have promise for clinical applications guiding both diagnosis and treatment.

There is evidence that treatment affects the samples 5hmC levels. All but two of our samples were from patients diagnosed when chemotherapy regimens were largely give in an adjuvant setting and are expected to be chemotherapy naïve. One glioblastoma sample taken at the time of autopsy timing of treatment was unknown. Aside from chemotherapy, children with CNS tumors are almost always given dexamethasone for tumor edema and this was reflected in our results. For instance, GREAT analysis of differentially hydroxymethylated regions in the tumor versus non-tumor comparison demonstrated the clustering of DHMRs in steroid response pathways. This is likely due to the treatment of most CNS tumors with dexamethasone in order to reduce edema and the risk of herniation. This relationship was present across all tumor subtypes. 5hmC has been associated with steroid-induced osteonecrosis and endometriosis, and our data provide further evidence of the connection between the medication and the epigenomic marker [62, 63].

## Conclusions

To our knowledge, we describe for the first time a genome-wide cytosine-specific analysis of 5hmC in three classes of childhood CNS tumors: embryonal, glioma, and ependymoma. Super-enhancer targeting by 5hmC in all three classes of tumors was identified, and genes commonly implicated in pediatric CNS tumors were differentially hypohydroxymethylated. We demonstrated that distinguishing methylation and hydroxymethylation is critical in identifying tumor-related epigenetic changes.

## Materials and methods

### Study population and samples

Pathologically confirmed fresh-frozen primary CNS tumor specimens from 27 unique individuals were identified; they included thirteen gliomas, eight ependymomas, and six embryonal tumors. All patients were treated at Dartmouth Hitchcock Medical Center. Samples were collected from patients who had provided consent for the use of tissues for research purposes as approved by the committee for the protection of human subjects (Institutional Review Board). Detailed information about each patient, including demographics, tumor histopathology, survival, time to recurrence, metastasis, chemotherapy and radiation regimens, was collected from the electronic medical record system. Pathologic re-review confirmed histopathologic tumor type and grade for all cases according to the 2016 WHO classification of CNS Tumors [3]. A table with complete patient information, including survival and treatment received is available (Additional file 1). Five patients had greater than one sample with two patients each having four samples available. Four fresh-frozen non-tumor brain specimens from children ranging in age from newborn to 11 years were selected as control tissues. Three tissues were from patients with epilepsy who underwent surgical resection and were used in our analyses. One tissue was excluded from analysis as it was from a non-immune hydrops fetalis subject at only 21 6/7 weeks gestational age.

### DNA extraction and modification

Tumor DNA was extracted from fresh frozen tumor tissue using DNeasy Blood and Tissue Kit (Qiagen) following the manufacturer’s instructions. Approximately, 1–15 mg of tumor tissue was used for DNA extraction. DNA was subjected to tandem bisulfite and oxidative bisulfite (OxBS) conversion using the TruMethyl OxBs module kit (Cambridge Epigenetix—Nugen), according to the TrueMethyl protocol M01481, Revision v2.

### *TERT* promoter and *H3F3A* sequencing

Tumor samples were sequenced for *TERT* Promoter (C2505, C280T) and *H3F3A* mutations status (K27M, G34) using PCR amplification and Sanger sequencing. ~ 10 ng of genomic DNA per sample was amplified (see Appendix for the list of primers). For *H3F3A*, OneTaq Hot Start 2 × Master Mix with standard buffer was used (NEB). PCR cycle parameters were as follows: denaturation at 95 °C for 30 s, followed by 35 cycles of 94 °C for 30 s, 48 °C for 30 s, 68 °C for 1 min, with the final extension at 68 °C for 5 min. Amplicons were visualized in Agarose Gel each time. For *TERT* Promoter, OneTaq Hot Start 2X Master Mix with GC buffer (NEB) was used. Cycle parameters were as follows: denaturation at 94 °C 30 s, followed by 35 cycles of 94 °C 30 s, 59 °C for 30 s, 68 °C for 1 min, with a final extension at 68 °C for 5 min. DNA was purified using the Qiagen PCR purification kit prior to visualization on an agarose gel. Primer sequences used can be found in Additional file 5.

### Cytosine modification measures

Illumina Human Methylation EPIC Beadchips were loaded with sample DNA in a randomized fashion with equal distribution of all tumor subtypes and non-tumor samples in each chip in order to eliminate batch effects. Beadchips were read using the Illumina iScan reader, and sample intensity data (IDAT files) was generated. Normalization and background correction from each of the BS and OxBS converted samples was performed using the *Funnorm* procedure available in the R/Bioconductor package *minfi* (version 1.30.0) [64]. Before analysis, we removed CpG sites on sex chromosomes using the updated Illumina EPIC annotation file (R/Bioconductor package *IlluminaHumanMethylationEPICanno.ilm10b4.hg19*) and SNP probes identified in the EPIC array [65]. To estimate methylated, hydroxymethylated, and unmethylated proportions of cytosine, we applied the OxyBS algorithm which takes bisulfite-treated data which includes both 5mC and 5hmC levels and approximates 5hmC signal intensity by subtracting oxidative bisulfite-treated data which provides only 5mC signal intensities [39]. Through this method, a total of 743,461 probes were left for analysis. Array data analysis was conducted in R version 3.6.1 [66].

### Statistical analyses

We identified and subset our data to the 5% (37,173/743,461) most highly hydroxymethylated CpG sites (high 5hmC loci), as determined by the median, across all tumor types. We performed Fisher’s exact tests for enrichment of these 37,173 consistently hydroxymethylated CpGs in CpG islands, shores, and shelves against the 743,461 CpG universe. For gene promoter, exon, and intron regions and regulatory elements, we used a Cochran-Mantel–Haenszel test to assess enrichment stratifying by probe type. The Phantom 5 enhancer annotation (available in the Illumina EPIC annotation file) was used to map CpGs in enhancer regions. Coordinates for super-enhancers were downloaded from the dbSuper database [43]. We examined the 486 genes in which these high 5hmC CpGs were enriched using the UCSC reference gene names in the Illumina annotation file. We selected for genes that had at least 10 CpGs represented in the EPIC array. We used the genomic coordinates of these high 5hmC CpGs in enriched genes as a query set of regions and submitted to Genomic Regions Enrichment of Annotations Tool (GREAT) analysis. We tested for enrichment against the background of the 743,173 CpGs in the EPIC array used in our analyses.

### Analysis of CpG-specific associations

Differential hydroxymethylation status between tumor and non-tumor brain tissue at the CpG loci in our data set was determined through multivariable linear models for microarray data (limma) [67]. Models were adjusted for subject age and sex and applied to 5hmC β-values. Benjamini–Hochberg correction was used to adjust for multiple testing. We examined whether there were differences between tumors and non-tumors in 5hmC levels within the top 5% most hydroxymethylated CpGs (37,173). We applied the same model to each tumor subtype and compared them to non-tumor samples in order to determine if the results changed when stratified.

To explore if the loss of tumor hydroxymethylation in high 5hmC loci was limited to specific gene sets, we selected CpGs that were differentially hypohydroxymethylated with negative log fold change (log-FC < 0) and FDR less than 0.1 (726 CpGs) and queried the GREAT software [68]. To test if these loci were associated with transcription factor binding sites or sites of histone modifications, we interrogated the ENCODE and Roadmaps database using the LOLA R/Bioconductor package [69]. We tested for enrichment in CpG islands, shores and shelves using a Fisher’s exact test against the 37,173 high 5hmC loci. We used the same universe to test for enrichment of DHMRs in regulatory regions and super-enhancers using a Cochran-Mantel–Haenszel test.

### Survival analysis

Recursively partitioned mixture model (RPMM) has been used for clustering DNA methylation and hydroxymethylation data to identify classes of tumors based on beta values [51]. Here, we applied RPMM to the top 10,000 most variable CpGs across tumors as determined by variance, and the resulting clustering solution contained two distinct clusters, defining a low and a high 5hmC cluster. A multivariable Cox proportional hazards model adjusting for age at diagnosis, sex, and tumor type was used to determine if cluster designation correlated with survival. Although one patient in our cohort had a diagnosis of glioblastoma, the sample was collected at autopsy and no clinical information was available. Therefore, due to the lack of follow-up, we excluded this patient from our survival analysis.

### Evaluation of 5hmC in methylation-based classification systems

Although the Capper classification system does not disclose the list of CpGs it uses to predict tumor type, we downloaded and processed the files from their experiment and isolated 31,476 with a S.D > 0.228 to capture an approximation of the 32,000 most variably methylated probes they state were used in their methods. In order to evaluate overlap of adult and pediatric tumors’ high 5hmC loci with the capper data set, we used IDAT files previously published experiments examining 5hmC levels in adult glioblastomas [13], reprocessed the files with ours and re-subset the complete dataset (encompassing both pediatric and adult data) to the CpGs with the top 5% hydroxymethylation levels. This included 19, 353 CpGs. Infinium Purify [52] was used to determine tumor purity from methylation levels and publically available tumor IDAT files [36] were downloaded to provide more robust predictions.

## Supplementary Information

**Additional file 1**. Includes a list of all samples, patient demographics, location and histopathologic diagnosis of the tumor, duration of clinical follow-up, treatments received as well as any known or tested mutations.

**Additional file 2**. *Gene List*: List of genes with a high proportion of represented probes being hyperhydroxymethylated. Gene name, number of probes for each gene represented in the EPIC array, number of probes that qualified as high 5hmC and the calculated percentage. *Results of GREAT analysis*: Results of submitting the coordinates of the high 5hmC loci to Genomic Regions Enrichment of Annotations Tool (GREAT) analysis. Terms associated with biologic process, cellular components and molecular function are included as well as *P* value and FDR Q-Values.

**Additional file 3**. *Great Analysis of DHMRs*: Results of submitting DHMR loci to GREAT analysis. *DHMR Genes*: Results of linear model testing for sites of differential hydroxymethylation between tumor and non-tumor samples. Data includes the associated genes, relation to island, chromosomes, logFC and adjusted *P* value.

**Additional file 4**. *Embryonal V NonTumor Ontologies*: Results of GREAT analysis of DHMRs from a linear model comparing embryonal and non-tumor samples. *Ependymoma V NonTumor Ontologies*: Results of GREAT analysis of DHMRs from a linear model comparing ependymomas and non-tumor samples. *Glioma V NonTumor Ontologies*: Results of GREAT analysis of DHMRs from a linear model comparing gliomas and non-tumor samples.

**Additional file 5**. List of primers and sequences used for sanger sequencing.

**Additional file 6**. Supplemental Figures and Tables.

## Data Availability

The pediatric tumor and non-tumor microarray data (IDAT files) were deposited at the Gene Expression Omnibus (GEO) under GSE152561 (http://www.ncbi.nlm.nih.gov/geo/).

## Abbreviations

5caC: 5-Carboxylcytosine, an oxidized form of 5-methylcytosine
5fC: 5-Formylcytosine, a derivative of 5-methylcytosine
5hmC: 5-Hydroxymethylation, an oxidized form of DNA methylation
5mC: 5-Methylcytosine, a methyl (CH3) group added to the fifth carbon in cytosine found commonly in DNA. Alterations in methylation are commonly found in cancer pathologies
CI: Confidence interval
CNS: Central nervous system which includes the brain and spinal cord
HR: Hazard ratio
IDH: Isocitrate dehydrogenase. A gene commonly mutated in adult glioblastomas
OR: Odds ratio
PCA: Principal component analysis. A statistical method that finds a line of best fit given a collection of points
TSS: Transcription start site

## Code availability

R code used for all analyses and figures in the paper can be found at https://github.com/Christensen-Lab-Dartmouth/PCNS_5hmC_06112020.
