# Supplementary material for: DNA 5-hydroxymethylcytosine in pediatric central nervous system tumors may impact tumor classification and is a positive prognostic marker
Source: Clin Epigenetics. 2021 Sep 19;13:176. doi: 10.1186/s13148-021-01156-9 (PMC8451154; doi:10.1186/s13148-021-01156-9)
Supplement: Supplementary file 6 — Additional file 6. Supplemental Figures and Tables. [file 13148_2021_1156_MOESM6_ESM.docx]

**Supplementary Table 1 SNPs associated with Tumors as Determined by Sanger Sequencing**

| Genetic Characteristics of Tumors | n (%) |
| --- | --- |
| H3F3A K27M | 0 (0) |
| H3F3AG34 | 0 (0) |
| TERT Promoter C280T | 0 (0) |
| TERT Promoter C250T | 0 (0) |
| rs2853669 | 17 (63) |
| rs35226131 | 1 (4) |
| rs35161420 | 1 (4) |

**Supplementary Table 1** Sanger sequencing was performed on all tumor samples. None were positive for the known *H3F3A* or *TERT* promoter mutations

**Supplementary Fig. 1 5hmC Levels Surrounding Transcription Start Sites Stratified by Tumor Type**

**
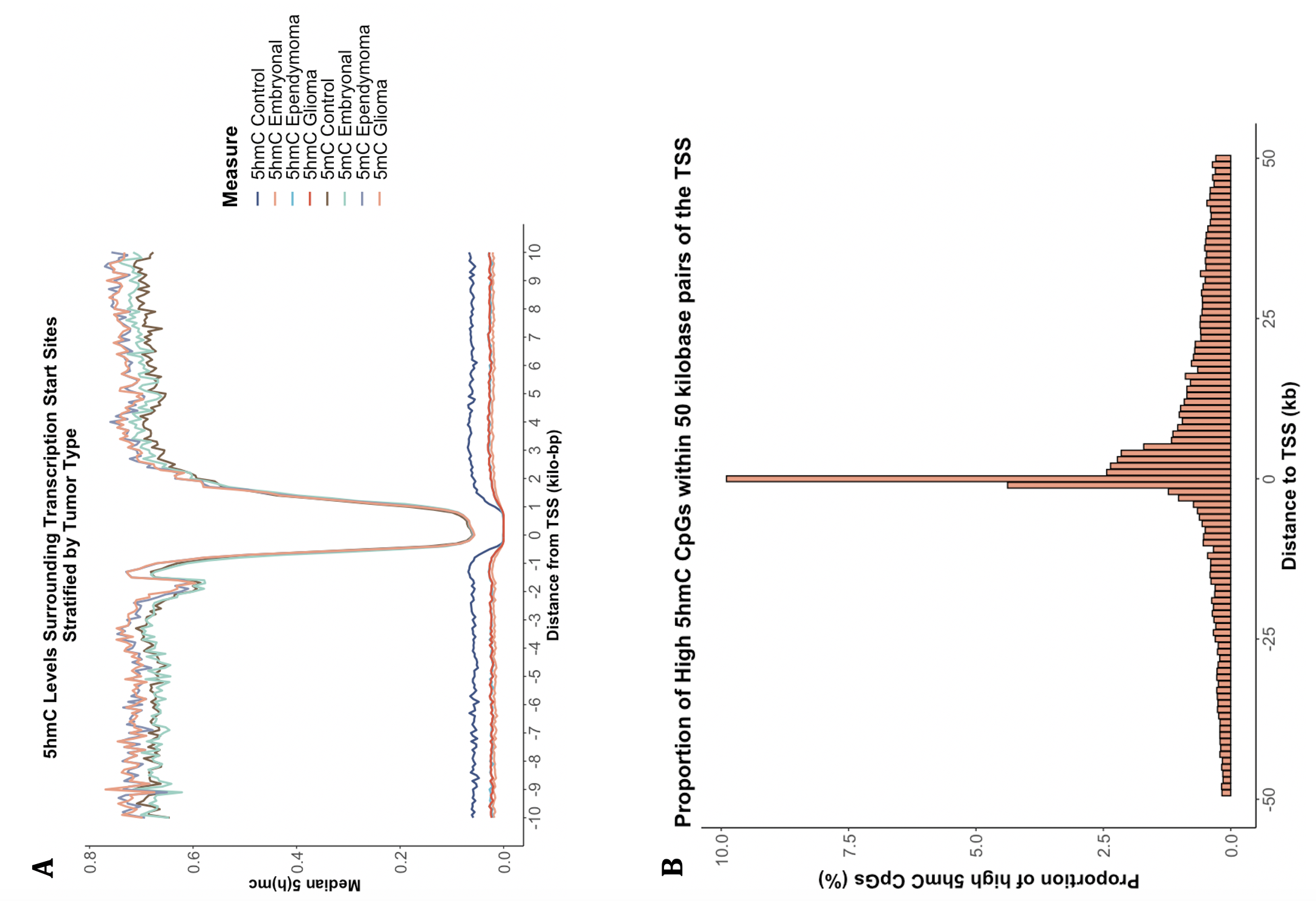
**

**Fig. 1 a)** Median 5hmC and 5mC levels of CpGs of each tumor subtype within 10,000 base pairs of the TSS. Methylation levels drop for all sample types (non-tumor, embryonal, ependymoma, and glioma) at the TSS. Gliomas and ependymomas were the most methylated of all tumor subtypes. There were no substantial differences in 5hmC levels between tumor subtypes. **b)** This plot represents the proportion of high 5hmC CpGs that fall within 50 kilobase pairs of the transcription sites. Approximately 21% of these sites are within 2,000 base pairs of the TSS. There is some asymmetry in their distribution with a greater tendency to be upstream closer to 5’ regions

**Supplementary Fig. 2: Variation of Median 5mC levels at High 5hmC Loci**


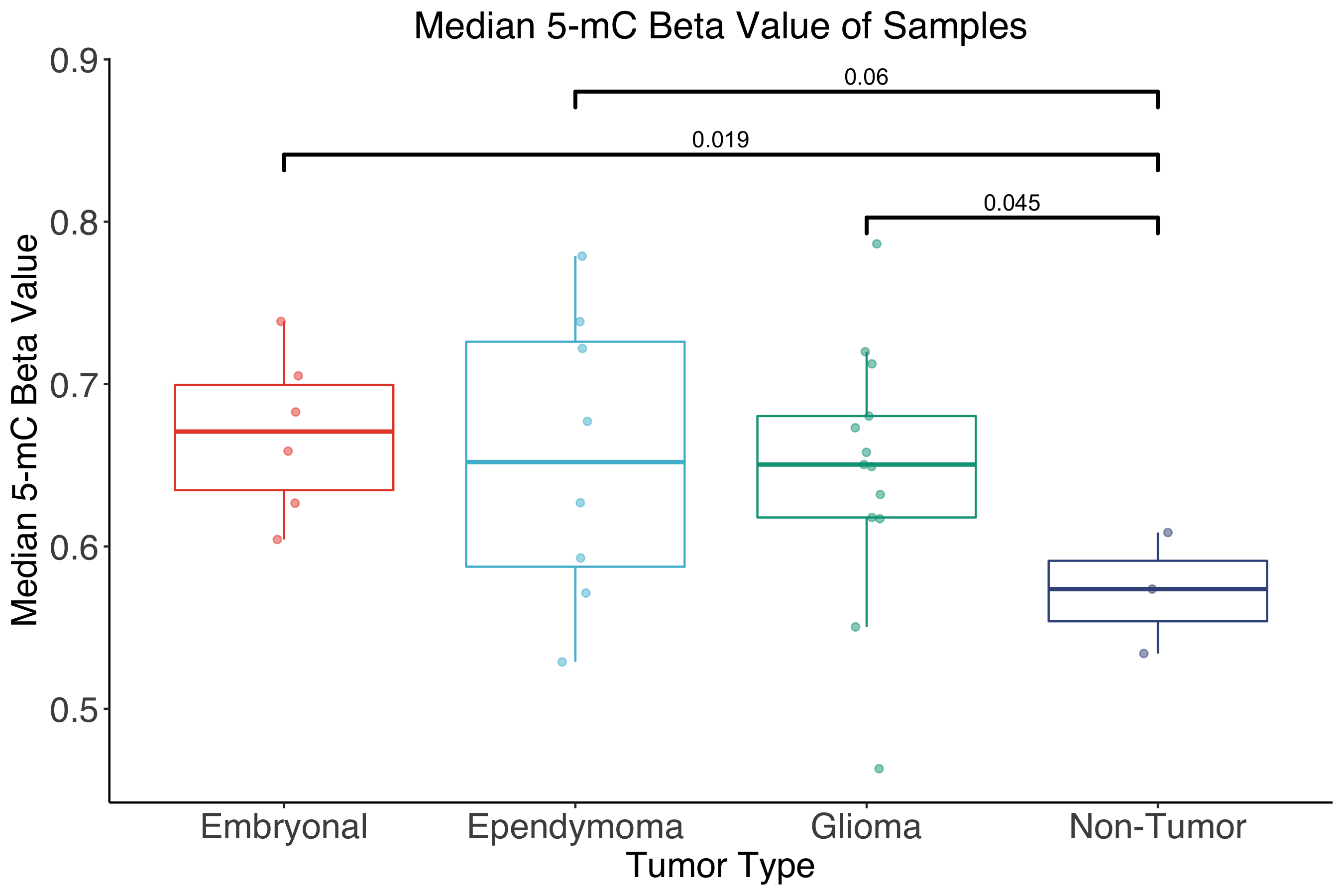


**Fig 2.** The figure above shows lower 5mC in non-tumors compared with tumor tissues among high 5hmC CpG sites, and relatively consistent 5mC among tumor subtypes.

**Supplementary Table 2 Association of High 5hmC Loci with CpG Islands**


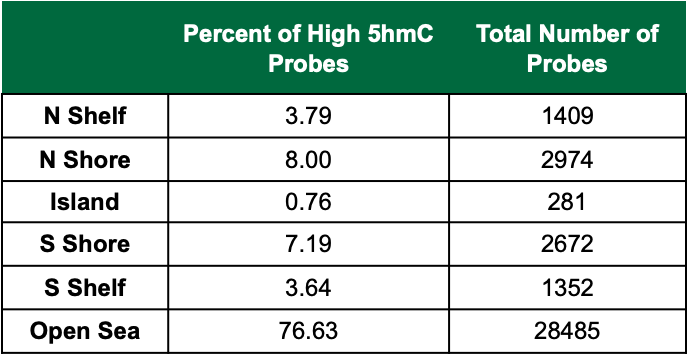


**Table 2** Table of percentage of all high 5hmC CpGs (n=37,173) found in relation to CpG islands

**Supplementary Table 3 Association of Differentially Hypohydroxymethylated Probes with CpG Islands**

**
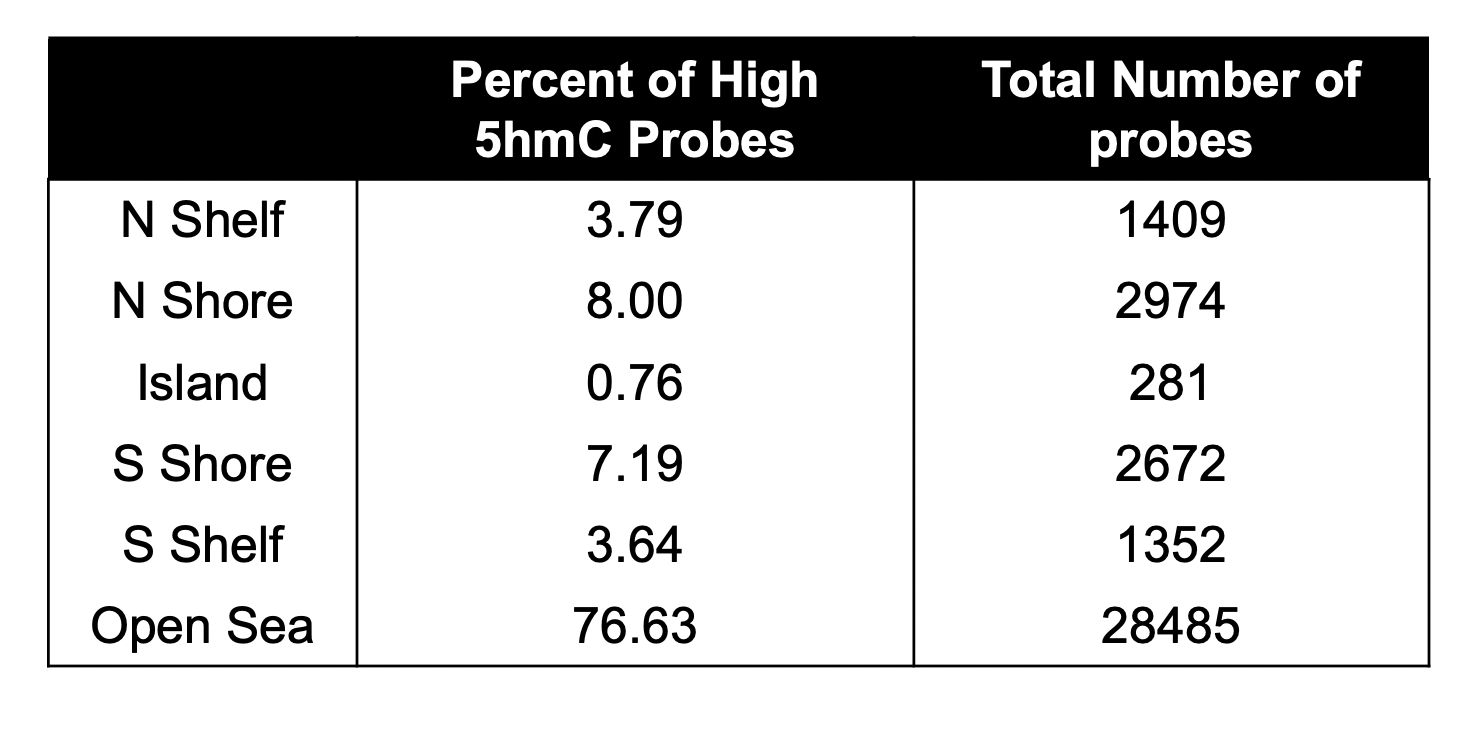
**

**Table 3** Table of proportions of differentially hydroxymethylated loci found in relation to CpG islands. 726 hypohydroxymethylated sites selected using a linear model comparing tumor and non-tumor high 5hmC loci

**Supplementary Fig. 3 Association of Differentially Hydroxymethylated Regions and Histone Modifications**


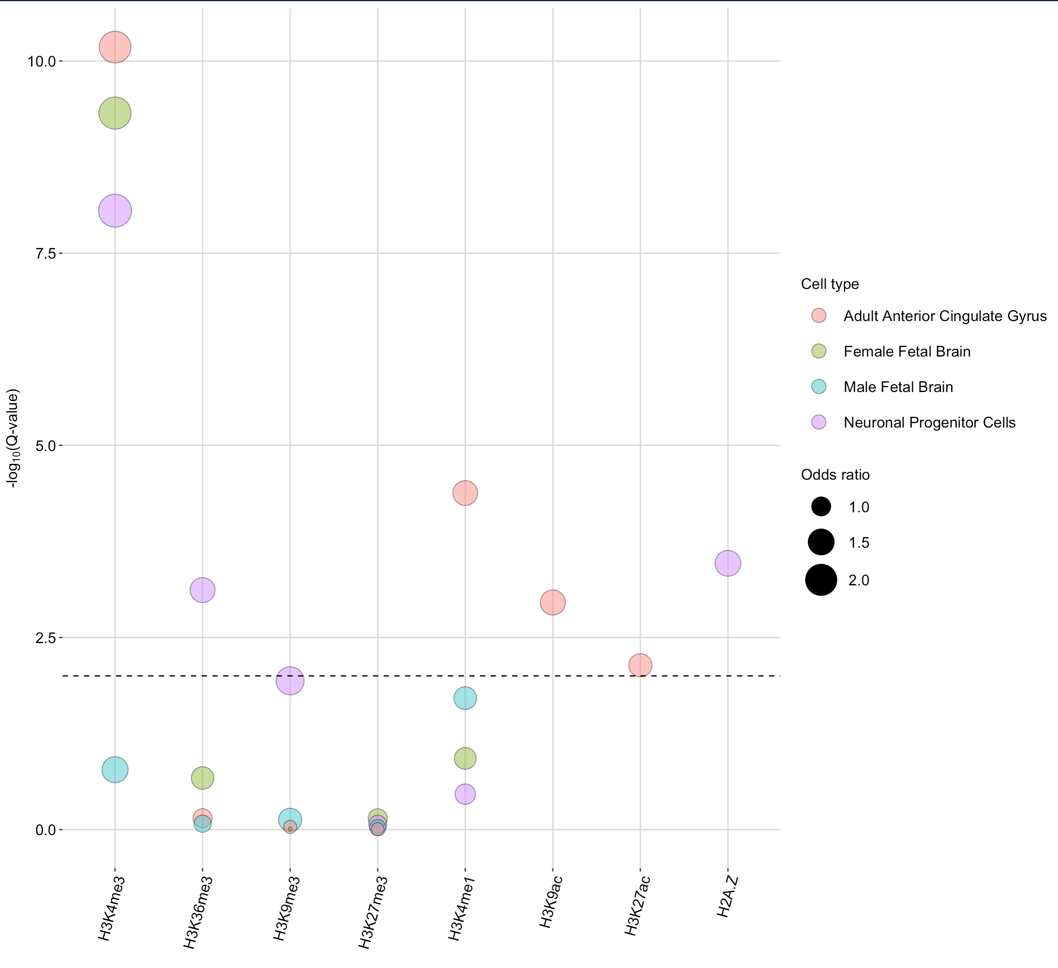


**Fig. 3** We used ChIP-Seq data from NIH Roadmap Epigenomics Project and coordinates from DHMRs to test overlap (LOLA) of the sequences of DHMRs with specific histone modifications. We chose fetal and adult neuronal cell lines for this analysis. Size in this image relates to odds ratio and color to the specific cell lines tested. The y-axis represents -log10 of p value adjusted for multiple testing. The dotted line represents a q value of 0.01. Regions that had loss of 5hmC were most strongly associated with repressive H3K4me3. However, activating modifications, H3K9ac and H3K27ac, and priming (H3K4me1) also met statistical significance

**Supplementary Fig. 4**

**
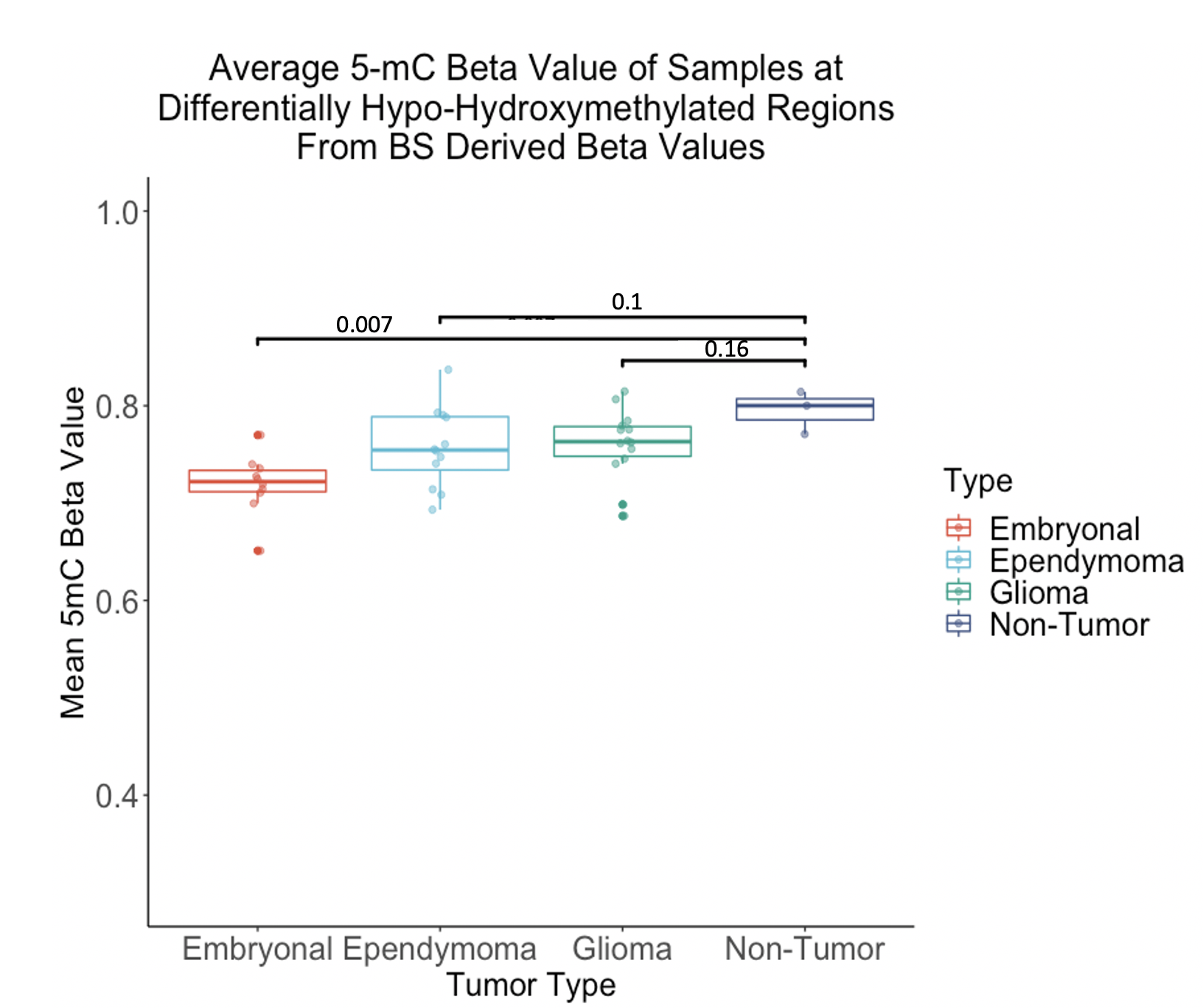
**

**Fig. 4** Average 5mC values of CpGs of all differentially hypohydroxymethylated regions of the high 5hmC regions (n =726) based on bisulfite treated samples which does not distinguish between 5mC and 5hmC. While OxBS derived data demonstrates that tumors are hypermethylated at loci that experience loss of 5hmC, bisulfite derived data does not distinguish between 5hmC and 5mC and instead shows a relative loss of methylation as compared to tumors. This data underscores the necessity to distinguish the two cytosine modifications

**Supplementary Fig. 5 Pairwise Comparison of Methylation Levels at DHMRs to All Measured Loci**


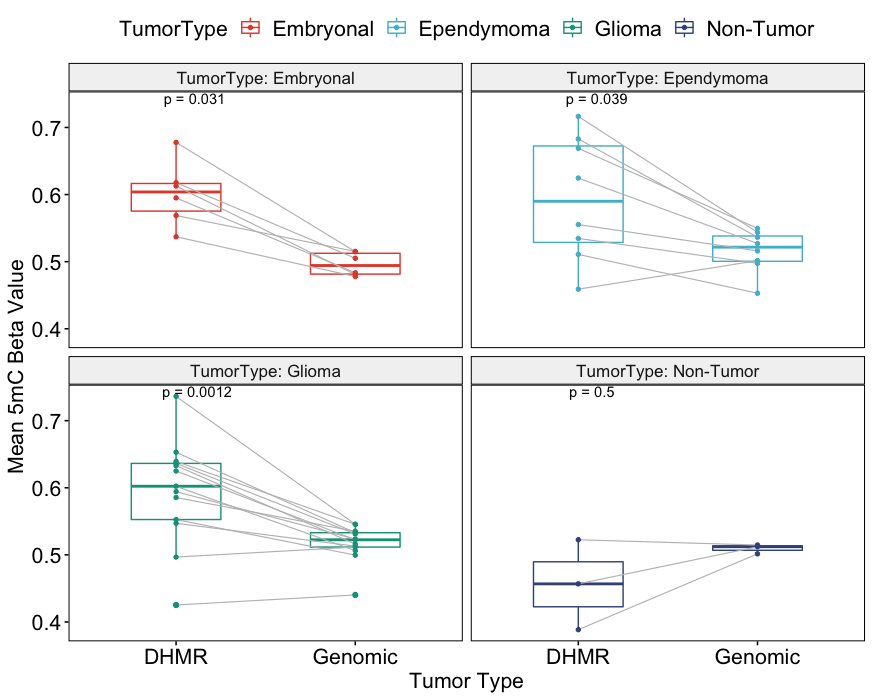


**Fig. 5** Pairwise comparison of 5mC levels at the differentially hypohydroxymethylated region (DHMR) loci to all other CpGs measured on the EPIC array. DHMRs have higher 5mC methylation levels in tumors, and these higher 5mC levels at DHMRs of non-tumor tissue have no significant differences from all other loci

**Supplementary Fig. 6 Genome Wide Differentially Hydroxymethylated Loci in Gliomas**


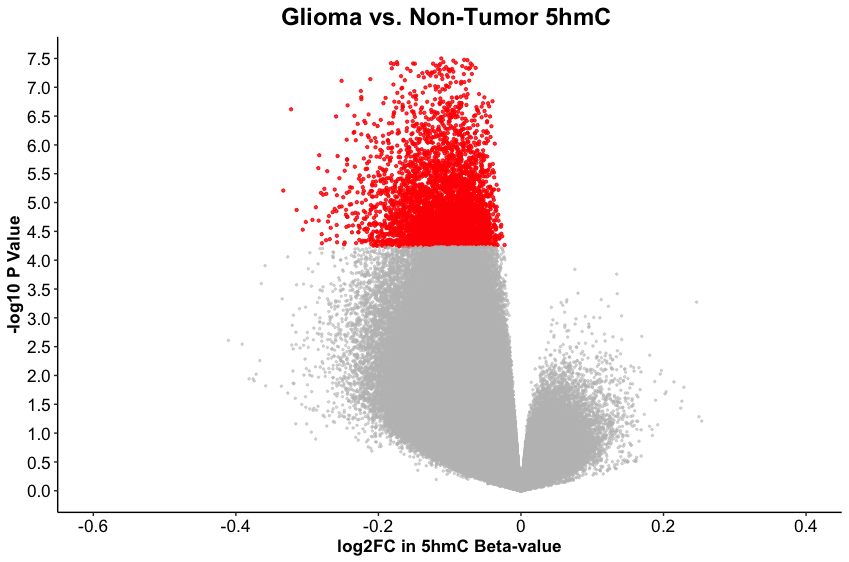


**Fig. 6** Though no differences arose when comparing gliomas to non-tumors within the high 5hmC CpGs, there was a question of whether this was due to an inherent bias of restricting our analysis to a small set of loci. When the list of CpGs was expanded to include all loci in the study and used in the same linear model, gliomas were found to be significantly differentially hypohydroxymethylated as compared to non-tumors with 25,147 CpGs having an adjusted p-value < 0.05

**Supplementary Table 4**

| **Cox Proportional Hazard Ratios for Survival** | | | |
| --- | --- | --- | --- |
| **Variable** | | **HR (95% CI)** | **P-value** |
| **Age** | | 0.94 (0.79-1.1) | 0.5 |
| **Sex** | |  |  |
|  | **Female** | 1.0 (referent) |  |
|  | **Male** | 1 (0.25-4.0) | 0.99 |
| **Grade** | |  |  |
|  | **Low** | 1.0 (referent) |  |
|  | **High** | 10.18 (1.04-99.8) | 0.046 |
| **RPMM Cluster** | |  |  |
|  | **High 5hmC** | 1.0 (referent) |  |
|  | **Low 5hmC** | 4.36 (0.53-35.6) | 0.17 |

| **Cox Proportional Hazard Ratios for Recurrence** | | | |
| --- | --- | --- | --- |
| **Variable** | | **HR (95% CI)** | **P-value** |
| **Age** | | 0.92 (0.79-1.1) | 0.5 |
| **Sex** | |  |  |
|  | **Female** | 1.0 (referent) |  |
|  | **Male** | 1 (0.78-2.6) | 0.69 |
| **Grade** | |  |  |
|  | **Low** | 1.0 (referent) |  |
|  | **High** | 7.73 (1.39-43.1) | 0.02 |
| **RPMM Cluster** | |  |  |
|  | **High 5hmC** | 1.0 (referent) |  |
|  | **Low 5hmC** | 2.96 (0.62-14.0) | 0.17 |

Running the model adjusting for grade (divided into low grade inclusive of grade 1 and 2 masses and high grade including grade 3 and 4) still maintained a high hazard ratio of 4.36 and a p-value that is <0.2 (see table below). It is certainly possible that 5hmC levels correlate with grade and that 5hmC can be a useful marker for tumor grade however future studies would need to be performed to validate this.

**Supplementary Fig. 7 Recurrence and Survival stratified by High and Low Grade Status**

**
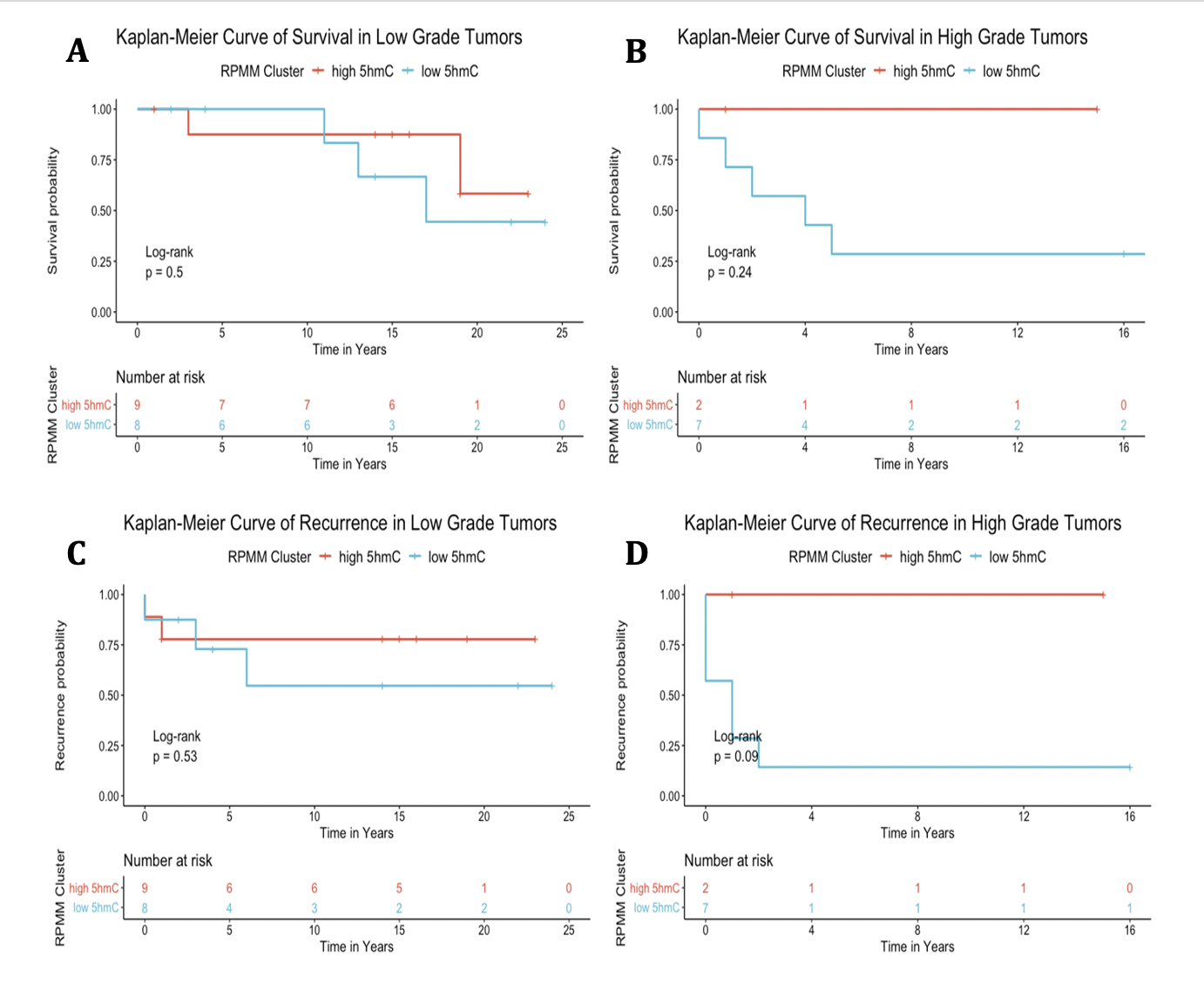
**

**Fig. 7** When stratified by grade recurrence and survival trends remain

**Supplementary Fig. 8**


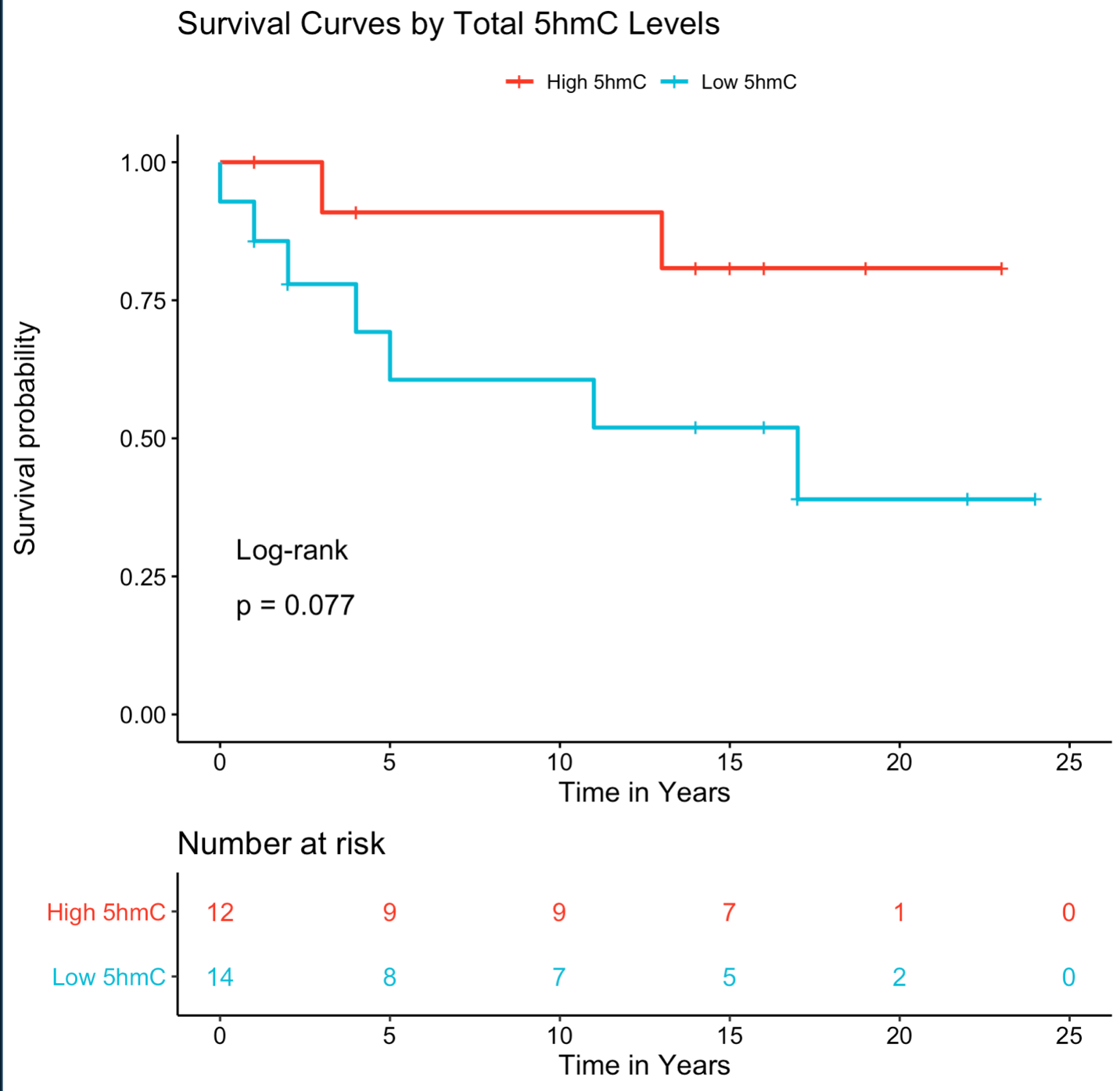


**Supplementary Fig. 8** Kaplan Meier survival curve of samples clusters determined by total 5hmC levels. Samples in the high 5hmC group had an average 5hmC across all CpGs analyzed greater than the 50^th^ percentile while those in the low 5hmC group were below that threshold. It follows a similar trend to the results from the RPMM modeling method though it did not meet statistical significance

**Supplementary Fig. 9 PCA analysis of the Most Variably Hydroxymethylated Loci
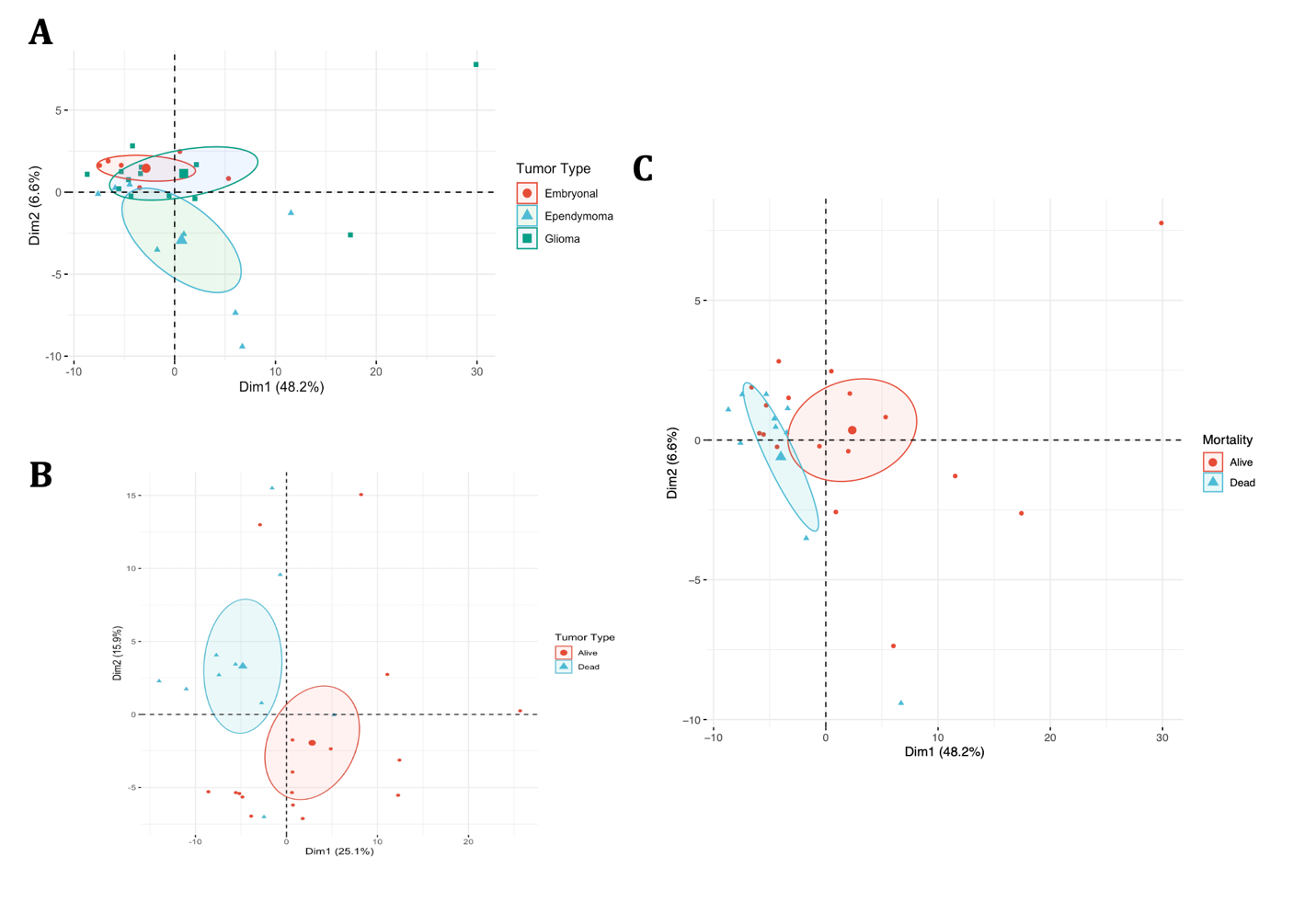
**

**Fig. 9** **a)** Principal component analysis of the top 10,000 most variably hydroxymethylated CpGs in tumors demonstrates clustering by diagnosis**. b)** These same loci are mapped with 5hmC values and the status of the patient at last follow up as alive or dead **c)** Using these loci with methylation beta values also yielded adequate clustering by survival status
